# Leptin enhances the efficacy of glucantime to modulate macrophage polarization toward the M1 phenotype in *Leishmania tropica*-infected macrophages

**DOI:** 10.1186/s13071-025-07004-6

**Published:** 2025-08-25

**Authors:** Alireza Keyhani, Abdollah Jafarzadeh, Iraj Sharifi, Ehsan Salarkia

**Affiliations:** 1 Leishmaniasis Research Center, Kerman University of Medical Sciences, Medical University Campus, Haft-Bagh Highway, Kerman, 7616913555 Iran; 2 Applied Cellular and Molecular Research Center, Kerman University of Medical Sciences, Kerman, Iran; 3 Department of Immunology, Medical School, Kerman University of Medical Sciences, Kerman, Iran

**Keywords:** Leishmaniasis, *Leishmania tropica*, Macrophages, M1/M2 macrophages, Leptin, Glucantime

## Abstract

**Background:**

Macrophages are essential immune cells during *Leishmania* infection, as their polarization toward M1/M2 phenotypes determines disease outcome. This study aimed to investigate the modulatory effects of leptin, alone and in combination with glucantime, on macrophage polarization in *Leishmania tropica* infection.

**Methods:**

Human THP-1-derived macrophages infected with *L. tropica* were treated with leptin (5 or 10 ng/ml), glucantime (100 or 200 μg/ml), or their combinations. The cytotoxic effects, parasite survival, reactive oxygen species (ROS), nitric oxide (NO) generation, and expression of M1/M2 acrophage-related parameters were evaluated using standard methods.

**Results:**

Both leptin doses significantly increased the expression of M1-associated markers (CD86, iNOS, SOCS3, miR-155) and pro-inflammatory cytokines (TNF-α, IL-12, IFN-γ) while decreasing M2-associated markers (CD206, ARG1, SOCS1, miR-146a) and anti-inflammatory cytokines (IL-4, IL-10, TGF-β). The leptin-glucantime combinations showed synergistic effects, shifting macrophage polarization toward the M1 phenotype more than either treatment alone. In particular, the combination of 10 ng/ml leptin with 100 μg/ml glucantime completely eliminated intracellular amastigotes and showed a superior selectivity index (17.66) compared to mono-treatment (leptin: 7.88; glucantime: 6.87).

**Conclusions:**

The findings indicate that leptin enhances the efficacy of glucantime against *L. tropica* by promoting M1 macrophage polarization. This presents a potential therapeutic approach that may lower conventional drug doses and associated toxicity while preserving or even improving treatment outcomes.

**Graphical Abstract:**

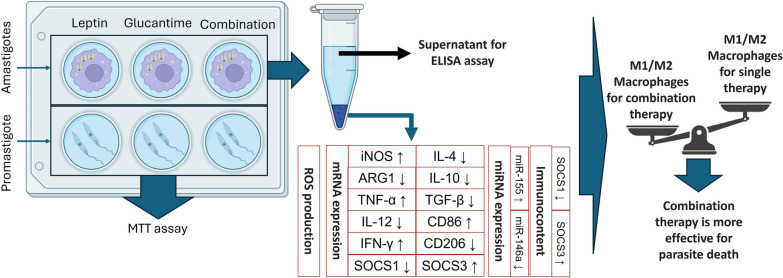

**Supplementary Information:**

The online version contains supplementary material available at 10.1186/s13071-025-07004-6.

## Background

Leishmaniasis is a major global health concern, particularly within the category of neglected tropical diseases transmitted by vectors [2]. Leishmaniasis presents with a diverse range of clinical presentations, including both classical and atypical forms. However, cutaneous leishmaniasis (CL), mucocutaneous leishmaniasis (MCL), and visceral leishmaniasis (VL) represent the three major clinical types [47]. While the precise burden of leishmaniasis is difficult to quantify, current data indicate a significant impact. It is estimated that 0.7–1.0 million new infections arise each year, affecting 12 million individuals globally. Tragically, this leads to an estimated 26,000 to 65,000 deaths annually within a population of 1 billion people considered at risk [41].

Cutaneous leishmaniasis (CL) is a prevalent and widespread infectious disease, posing significant public health challenges in approximately 70 countries. Nearly 95% of global CL cases occur in tropical regions, including the Americas, the Mediterranean, the Middle East, and Central Asia. The World Health Organization (WHO) Eastern Mediterranean Region bears the heaviest burden, accounting for 70% of new cases worldwide. In 2017, a substantial majority (> 95%) of CL cases were reported in six countries: Afghanistan, Syria, Algeria, Iran, Colombia, and Brazil [4, 5]. CL is a skin infection transmitted through the bites of female sandflies. The disease can cause skin sores on exposed areas of the body. Although it may heal naturally, it can lead to lifelong scarring, noticeable disfigurement, severe disabilities, and significant psychological impacts, including depression and social isolation [39].

Within the immune system, macrophages are essential in defending against *Leishmania* parasites, serving as both host cells and active contributors to the immune response. The outcome of *Leishmania* infection is determined by the status of macrophage activation, as they play a dual role during leishmaniasis [27, 36]. The polarization of macrophages into the pro-inflammatory M1 phenotype is related to resistance against infection, whereas the anti-inflammatory M2 phenotype is associated with increased susceptibility [10].

Macrophages are functionally categorized into two subgroups. M1 macrophages are primarily induced by LPS, IFN-γ, TNF-α, and GM-CSF [21, 40, 48]. M1 macrophages release a range of pro-inflammatory cytokines, such as TNF-α, IL-1, IL-6 and IL-12, playing a key role in defense against intracellular pathogens, such as the *Leishmania* parasite. M1 macrophages express markers such as CD68, CD86, and iNOS [30, 33]. Various stimuli, such as IL-10, TGF-β, IL-4/IL-13, M-CSF, and vitamin D3, induce M2 macrophage polarization [8, 24]. M2 macrophages typically secrete anti-inflammatory cytokines such as IL-10 and TGF-β, which are essential for tissue repair and resolution of inflammation [21]. M2 macrophages express markers such as CD163, CD206, and Arg-1 [21, 46].

Various factors, in particular microRNAs and the suppressors of cytokine signaling (SOCS) molecules, also play a key role in the macrophage polarization [19, 20]. High expression of SOCS3 is associated with M1 macrophage polarization, while SOCS1 facilitates the resolution of inflammation by enhancing M2 functions [37, 44, 45]. Some miRNAs (such as miR-127, miR-155, and miR-125b) can promote M1 polarization, while other miRNAs (miR-124, miR-223, miR-132, and miR-146a) can induce M2 polarization [15, 22, 28].

Deviation of the balance of macrophages towards the M2-type causes the infection to progress, which requires correction [40]. The adipocyte-derived hormone leptin can exert powerful immunoregulatory impacts on various cell types of the innate and adaptive immune response [34]. Leptin plays a significant role in the immune response during *Leishmania* infection. Lower serum leptin levels were associated with more severe cases of VL, suggesting that leptin may serve as a prognostic marker. In malnourished VL models, exogenous leptin restored T cell-mediated immunity [31]. Leptin treatment in *Leishmania donovani*-infected mice reduced splenic parasite burden and induced a Th1 immune response, characterized by elevated production of IFN-γ and IL-12 [31]. Furthermore, in vitro studies in mice have revealed that leptin, when combined with low doses of miltefosine (an anti-leishmanial drug), effectively inhibits the growth of *L. donovani* within macrophages [42]. However, the modulatory effects of leptin on *Leishmania*-infected macrophages and their polarization and activity have not been evaluated yet. Therefore, this study was conducted to investigate the modulatory effects of leptin, alone or in combination with glucantime, on the expression of some M1/M2-related parameters (including cytokines, markers, miRNAs and SOCS molecules) in *Leishmania tropica*-infected macrophages to clarify its potential.

## Methods

### Parasite culture

Promastigotes of *L. tropica* (MHOM/IR/75/Mash2), a common Iranian strain, were cultured in a standard RPMI-1640 medium supplemented with 10% fetal bovine serum (FBS) and 100 IU/ml penicillin/streptomycin. The cultured parasites were then maintained at an incubation temperature of 25 °C.

### Monocyte culture and cell differentiation

THP-1 cells (a human monocytic cell line) were cultured in 25 cm^2^ sterile culture flasks using RPMI 1640 complete medium supplemented with 10% FBS and 100 IU/ml penicillin-streptomycin. The cells were cultured at a density of 1 × 10⁶ cells/ml and incubated under controlled conditions at 37 °C with 5% CO₂. The culture medium was replaced every 2 days.

For differentiation into macrophages, THP-1 cells were seeded into 24-well plates at a concentration of 5 × 10^5^ cells/ml in complete medium. The cells were then treated with 100 ng/ml phorbol 12-myristate 13-acetate (PMA) (Sigma-Aldrich, USA) and incubated at 37 °C with 5% CO_2_ for 48–72 h. PMA treatment induced macrophage differentiation, characterized by morphological changes such as increased adherence to culture plates [16].

### Determining leptin and glucantime-related cytotoxicity effects (CC50 and IC50) on macrophages and parasites

The effects of leptin and glucantime on the viability of *L. tropica* promastigotes and macrophages were assessed using the 3-(4,5-dimethylthiazol-2-yl)-2,5-diphenyltetrazolium bromide (MTT) assay. This colorimetric assay measures the reduction of MTT by metabolically active cells, leading to the formation of a purple formazan product. After parasite/macrophage treatment with leptin or glucantime, 20 µl of MTT solution (5 mg/ml solution in 1% PBS) was added to each well and incubated for 4 h. Following incubation, 100 µl of dimethyl sulfoxide (DMSO) was added to dissolve the formazan crystals. The absorbance of each well was then measured at 570 nm using an ELISA reader (BioTek-ELX800). The absorbance values correlate directly with the number of viable cells in each well, reflecting the impact of treatments on cell viability. The concentration of a substance that reduces cell viability by 50% is called the cytotoxic concentration 50% (CC50) [32].

IC50 of leptin and glucantime against *L. tropica* promastigotes was determined by seeding promastigotes at a density of 2 × 10^5^ cells/well in a 96-well flat-bottom microtiter plate containing RPMI 1640 medium. Graded leptin concentrations, including 2.5, 5, 10, and 20 ng/ml, along with the reference standard drug glucantime (IC50 = 1 × 10^5^ ng/ml) were introduced into each well containing 2 × 10^5^ cells and incubated at 25 °C for 72 h. Following the initial incubation, 5 mg/ml MTT solution was added to the promastigotes in each well. The plate was then incubated for 3 h at 25 °C to allow formazan crystal formation. These crystals were subsequently dissolved by adding acidified isopropanol and incubating for 30 min at 37 °C. The absorbance in each well was then read at 570 nm using a BioTek-ELX800 ELISA reader [3]. The susceptibility of *L. tropica* promastigotes to leptin and glucantime was determined using the MTT colorimetric assay. The inhibitory concentration (IC50) value was defined as the drug concentration causing a 50% reduction in parasite survival.

To determine the IC50 of leptin and glucantime against *L. tropica* amastigotes, PMA-induced macrophages were cultured in RPMI-1640 medium supplemented with 10% FCS and 100 µg/ml penicillin-streptomycin at 37 °C with 5% CO_2_ atmosphere. PMA-induced macrophages were incubated with *L. tropica* promastigotes at a ratio of 10 parasites per macrophage. The macrophages were incubated at 37 °C for 6 h and then washed twice to eliminate any free parasites [17]. Different leptin concentrations (2.5, 5, and 10 ng/ml) were then added, and the flasks were incubated at 37 °C with 5% CO_2_ for 72 h. Each experiment was performed in triplicate. After that, the microscopic slides were prepared from each cell suspension and subsequently stained with Giemsa. The percentage of infected macrophages and the number of intracellular parasites per infected macrophage were assessed to determine the IC50 of leptin. To assess the therapeutic potential of each compound, its selectivity index (SI) was calculated. The SI is determined by dividing the CC50 in macrophages by the concentration of anti-*Leishmania* activity (IC50): [SI = CC50/IC50]. A higher SI value indicates a greater selectivity of the compound for the parasite over host cells, suggesting a better therapeutic potential [43].

### Detecting reactive oxygen species produced by parasite-infected macrophages

To assess the generation of ROS, macrophages infected with *L. tropica* were treated with 5 and 10 ng/ml leptin, 100 and 200 μg/ml glucantime, or a combination of leptin and glucantime, including 100 μg/ml glucantime combined with 5 or 10 ng/ml leptin. Untreated infected macrophages were also considered as control cultures. Following a 72-h incubation at 37 °C, the cells were washed with pre-warmed PBS and centrifuged at 800 g for 10 min. The cells were then resuspended in PBS containing the cell-permeable probe CM-H2DCFDA at a final concentration of 5 µM and incubated at 37 °C for 30 min. After re-washing with PBS, the fluorescence intensity of the cells was detected using flow cytometry with a blue laser excitation. Signals were recorded in the FL1/FITC channel to quantify ROS levels in both the treated and control macrophages [9]. The amount of ROS production by treated macrophages was calculated relative to that produced by untreated infected macrophages and expressed as a ratio.

### Detecting nitric oxide produced by parasite-infected macrophages

*Leishmania tropica*-infected macrophages were treated with 5 and 10 ng/ml leptin, 100 and 200 μg/ml glucantime, or combinations of leptin and glucantime, including 100 μg/ml glucantime combined with 5 ng/ml leptin or 100 μg/ml glucantime combined with 10 ng/ml leptin. A culture of untreated macrophages, which were stimulated with LPS (10 μg/ml), was considered positive control cells. The untreated and treated infected macrophages were incubated at 37 °C for 72 h, and then their supernatants were harvested at specific time points to assess the NO production using the Griess test. Briefly, the supernatants were mixed with Griess reagent (containing 1% sulfanilamide, 0.1% naphthylethylendiamine dihydrochloride, 3% H3PO4 in H_2_O) (all reagents obtained from Sigma, USA). The mixture of supernatant and Griess reagent was then incubated at room temperature for 5 min. Ultimately, the absorbance of controls and treated macrophages was measured at 570 nm[13].

### Determining expression of M1/M2-related molecules

To determine the effect of drug treatment on expression of M1/M2-related molecules (cytokines, surface markers, miRNAs, and SOCS molecules), *L. tropica*-infected macrophages were treated with leptin, glucantime, or their combinations at the mentioned doses. The mRNA expression of miRNAs, cytokines, surface markers, and SOCS molecules was assessed at 24 h after the beginning treatment. RNA extraction was done from treated and untreated macrophages at 24 h using YTzol Pure RNA (Yekta Tajhiz Azma, Iran) according to the manufacturer’s instructions. The quality and concentration of the extracted RNA were assessed by determining their absorbance at 260 and 280 nm using a NanoDrop system (BioTek-Synergy/HTX-Multi-mode reader). The extracted total RNA was then converted to complementary DNA (cDNA) synthesis using a cDNA synthesis Kit (Yekta Tajhiz Azma, Iran), according to the manufacturer’s instructions. Quantitative real-time PCR (qPCR) was used to determine the mRNA expression of M1/M2-related molecules, including cytokines (TNF-α, IL-10, IL-4, IFN-γ, TGF-β, and IL-12), CD markers (CD86 and CD206), and SOCS molecules (SOCS1 and SOCS3) using specific primers (Table 1). Each reaction was performed in triplicate and contained 2 µl of cDNA (200 ng), 10 µl of SYBR® Green master mix Kit™ (Ampliqon, Denmark), 1 µl of each primer (10 pmol), and 7.2 µl of double-distilled water. A real-time PCR system (Rotor-Gene Q, Qiagen, USA) was used to run the reactions according to the following program: one initial cycle at 95 °C for 15 min, followed by 40 cycles at 95 °C for 20 s and 60 °C for 60 s. The GAPDH was utilized as an internal control, and the mRNA expression of the interested genes was calculated using the 2^^−ΔΔCt^ method. The real-time PCR products were electrophoresed on agarose gel, and their melting curve was also performed at a temperature range from 70 °C to 95 °C, with a 1 °C increment in temperature, to determine the specificity of the reactions.

**Table 1:** The sequences of the primers used for quantitative PCR extracting from the Primer Bank (http://pga.mgh.harvard.edu/primerbank/)

Genes	Forward primers 5ˈ- 3ˈ	Reverse primers 5ˈ- 3ˈ	PCR product (bp)
ARG-1	TGGACAGACTAGGAATTGGCA	CCAGTCCGTCAACATCAAAACT	102
TGF-β	CAATTCCTGGCGATACCTCAG	GCACAACTCCGGTGACATCAA	86
TNF-α	GAGGCCAAGCCCTGGTATG	CGGGCCGATTGATCTCAGC	91
SOCS1	TTTTCGCCCTTAGCGTGAAGA	GAGGCAGTCGAAGCTCTCG	107
SOCS3	CCTGCGCCTCAAGACCTTC	GTCACTGCGCTCCAGTAGAA	99
IL-12	TGCCCATTGAGGTCATGGTG	CTTGGGTGGGTCAGGTTTGA	101
IL-4	CGGCAACTTTGTCCACGGA	TCTGTTACGGTCAACTCGGTG	111
IL-10	GACTTTAAGGGTTACCTGGGTTG	TCACATGCGCCTTGATGTCTG	112
IFN-γ	TCGGTAACTGACTTGAATGTCCA	TCGCTTCCCTGTTTTAGCTGC	93
CD206	CTACAAGGGATCGGGTTTATGGA	TTGGCATTGCCTAGTAGCGTA	105
CD86	CTGCTCATCTATACACGGTTACC	GGAAACGTCGTACAGTTCTGTG	133
GAPDH	ACAACTTTGGTATCGTGGAAGG	GCCATCACGCCACAGTTTC	101
UBC	CTGGAAGATGGTCGTACCCTG	GGTCTTGCCAGTGAGTGTCT	117

The sequences for microRNAs miR-146a and miR-155 were obtained from the miRbase database (http://www.mirbase.org/). Forward primers were designed to target the six nucleotides located at the 3’-end of the mature miRNA within the stem-loop structure, as this region is crucial for target binding. Primer and probe melting temperatures (Tm) were optimized using Gene Runner software. Secondary structures of stem loops were verified using Gene Runner. Specificity was confirmed by performing real-time PCR using cDNA generated from the stem-loop structures of each miRNA. Relative expression (fold change) was calculated by comparing the cycle threshold (Ct) values of each microRNA to those of the U6 small nuclear RNA as a housekeeping gene (Table 2).

**Table 2:** Stem loop, primers, and probe used for specific synthesis of cDNA and performing quantitative PCR regarding miRNA amplification

Primer types	miRNAs	Sequence 5 → 3
Stem loop	miR-155-5p	GTATGCTGCTACCTCGGACCCTGCTTAGTGCCATGCCTGCCATCGAGCAGCATAC AACCCC
	miR-146a	GTATGCTGCTACCTCGGACCCTGCTTAGTGCCATGCCTGCCATCGAGCAGCATAC AACCCA
	U6	GTATGCTGCTACCTCGGACCCTGCTTAGTGCCATGCCTGCCATCGAGCAGCATACAAAAATATGG
Forward	miR-155-5p	GCGCCTGAGAACTGAATTCCATGGGTT
	miR-146a	GCGCCTGAGAACTGAATTCCATGGGTT
	U6	GCAAGGATGACACGCAAATTCG
Probe	TaqMan probe	AGTGCCATGCCTGCCATCGAGC
Reverse	Universal	GCTGCTACCTCGGACCCT

### Determining parasite-infected macrophages expressing SOCS1 and SOCS3 proteins

Immunofluorescence staining was performed to determine the effect of drug treatment on protein expression of SOCS1 and SOCS3 molecules in *L. tropica*-infected macrophages. The untreated and treated parasite-infected macrophages were fixed in 4% paraformaldehyde for 20 min at room temperature. Non-specific antibody binding was blocked by incubation in PBS containing 0.1% Triton X-100 and 1% FBS for 20 min at room temperature. For immunofluorescence labeling, cells were incubated overnight at 4 °C with the following primary antibodies: a rabbit polyclonal antibody against SOCS1 (Elabscience, E-AB-32936, USA) and a mouse monoclonal antibody against SOCS3 (Santa Cruz Biotechnology, 6A463, USA). The control group was incubated with PBS only. After three washes with PBS, cells were incubated for 1 h at room temperature in the dark with the secondary antibodies: CY3-conjugated Goat Anti-Mouse IgG (H + L) and FITC-conjugated Goat Anti-Rabbit IgG(H + L) (Elabscience, USA). Images were acquired using an Olympus BX50 fluorescence microscope, and fluorescence intensity was quantified using ImageJ software.

### Measuring production of pro- and anti-inflammatory cytokines and SOCS molecules by parasite-infected macrophages

The ELISA method was used to determine the effect of drug treatment on cytokine production in *L. tropica*-infected macrophages. The supernants were collected from untreated and treated parasite infected-macrophages at 72 h, and the concentrations of TNF-α, IL-10, IL-4, IFN-γ, TGF-β, SOCS1, SOCS3, and IL-12 were determined by commercial ELISA kits, including human TNF-α (E-EL-H0109, Elabscience, USA), IL-10 (KPG-HIL-10, Karmania Pars Gene, IRAN), IFN-γ (E-EL-H0108, Elabscience, USA), IL-4 (KPG-HIL-4, Karmania Pars Gene, IRAN), TGF-β (E-EL-0162, Elabscience, USA), SOCS1 (MBS2021770, MyBioSource, USA), SOCS3 (MBS703435, MyBioSource, USA), and IL-12 (KPG-HIL-12, Karmania Pars Gene, IRAN) ELISA kits following the manufacturer’s instructions.

### Statistical analysis

Datasets were statistically analyzed using GraphPad Prism version 8.0.2 software. One-way analysis of variance (ANOVA) was employed to determine significant levels for ROS measurement, nitric oxide and arginase assays, and qPCR and cell cycle analyses. The IC_50_ values (50% inhibitory concentrations) were analyzed in SPSS using the probit test. A *t*-test was utilized to identify differences in IC_50_ values between the two stages of the *L. tropica* life cycle. The selectivity index (SI) is the ratio of the cytotoxic concentration of a sample to its effective bioactive concentration. It was computed as CC_50_ for peritoneal macrophage cells/IC_50_ ≥ 10, indicating non-toxicity. The data obtained from triplicate experiments were reported as the mean ± standard deviation (SD). Statistical significance was considered at *P* < 0.05.

## Result

### The cytotoxic effects of leptin and glucantime on *Leishmania* promastigotes and amastigotes

**Fig. 1:**
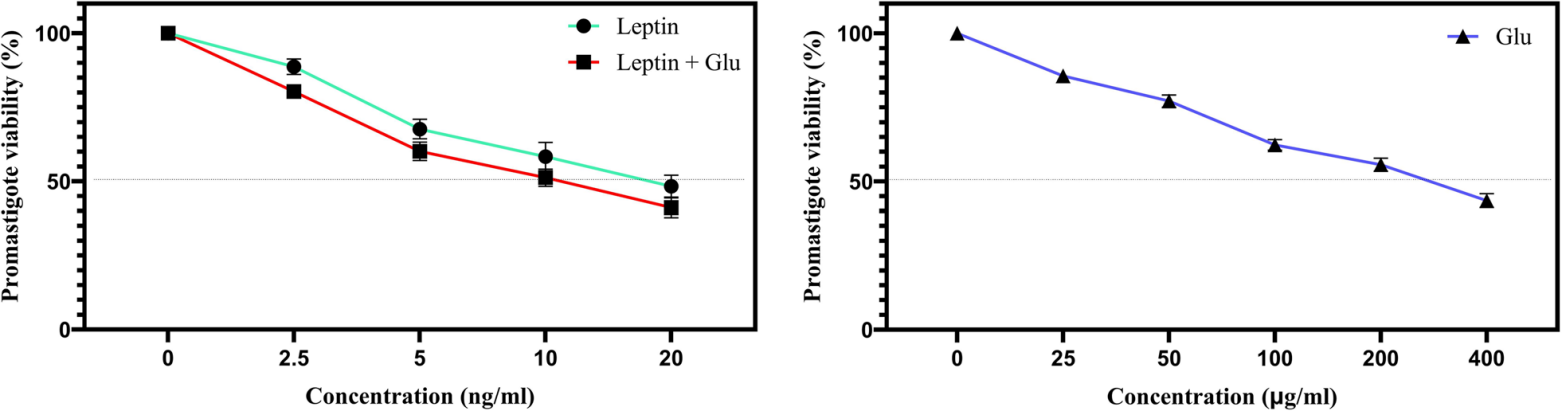
The cytotoxic effect of the different doses of leptin, glucantime, and their combination (Lep + Glu) on the *Leishmania tropica* promastigote. To determine the IC50 of leptin and glucantime against promastigotes, 2 × 10^5^ parasites were incubated with graded concentrations of leptin (0, 2.5, 5, 10, and 20 ng/ml) and glucantime (0, 25, 50, 100, 200, and 400 µg/ml) for 72 h. After adding the MTT solution and allowing formazan crystals to form, they were dissolved by adding acidified isopropanol. Finally, the absorbance in each well was measured at 570 nm using an ELISA reader system. IC50 was defined as the drug concentration that reduces parasite survival by 50%. The experiments were performed in five sets, and the results are expressed as mean ± SD

The study found that both leptin (Lep) and glucantime (Glu) singularly inhibited the growth of promastigotes in a concentration-dependent manner, with IC50 values of 16.57 and 239.34, respectively. Importantly, the combination of Lep and Glu exhibited a significantly enhanced inhibitory effect on the parasite compared to each drug alone or the negative control (IC50 = 10.41; *P* < 0.001). This suggests that the combined treatment may have a synergistic effect and may be more effective than each drug alone in controlling promastigote multiplication. Treatment with leptin, glucantime, or their combination (Lep + Glu) significantly increased the mortality rate of *L. tropica* promastigotes compared to the untreated control group (UC) (*P* < 0.001). This effect was observed across a range of drug concentrations (Fig. 1).

Table 3 illustrates the effect of the different doses of leptin and glucantime, or their combination, on the number of intracellular amastigotes assessed by the mean number of intracellular organisms at various concentrations. Notably, the combination of Lep (10 ng/ml) and Glu (100 mg/ml) completely eliminated the organisms. IC50 values were subsequently calculated.

**Table 3:** The effect of leptin and glucantime, or their combination (Lep + Glu), on the *Leishmania tropica* amastigote

Types of cultures	No. of intracellular amastigotes (mean ± SD)	*P* values*	*P* value**
Untreated control	31.6 ± 2.1	–	–
Lep-2.5	26.6 ± 1.8	< 0.001	< 0.001
Lep-2.5 + Glu-100	14.3 ± 0.7	< 0.001	
Lep-5	19.7 ± 0.4	< 0.001	< 0.001
Lep-5 + Glu-100	7.3 ± 0.3	< 0.001	
Lep-10	13.1 ± 0.2	< 0.001	< 0.001
Lep-10 + Glu-100	0.0 ± 0.0	< 0.001	
Lep-20	4.2 ± 0.1	< 0.001	< 0.001
Lep-20 + Glu-100	0.0 ± 0.0		
Glu 50	23.3 ± 1.1	< 0.001	–
Glu-100	16.8 ± 0.8	< 0.001	–
Glu-200	8.4 ± 0.6	< 0.001	–
Glu 400	0.0 ± 0.0	< 0.001	–

^*^P values in compared with untreated control culture

^**^P values regarding differences between Lep-treated culture and Lep + Glu-treated culture

### Calculation of selectivity index (SI) of Glu, Lep, and their combinations to determine safety

Lep, Glu, and the combinations of Lep + Glu demonstrated significant anti-leishmanial activity against intramacrophage amastigotes, as evidenced by their respective IC50 values. IC50 values were 108.9 µg/ml, 7.81 ng/ml, and 2.47 ng/ml for Glu, Lep, and the combination of Lep + Glu, respectively. Although all three regimens displayed a safe selectivity index (SI), the combination of leptin and glucantime exhibited a superior safety profile (Table 4). The formula of SI indicates a synergistic effect and highlights the safety of this mixture.

**Table 4:** Anti-leishmanial activity and selectivity index of leptin and glucantime

Treatment types	IC50 on promastigotes	IC50 on amastigotes	CC50 on macrophages	Selective index
Glu (μg/ml)	239.34 ± 0.17	108.9 ± 0.09	749.18 ± 0.044	6.87
Leptin (ng/ml)	16.57 ± 0.3	7.81 ± 0.05	61.53 ± 0.079	7.88
Leptin + Glu	10.41 ± 0.47	2.47 ± 0.026	43.62 ± 0.2	17.66

*IC50* half-maximum inhibitory concentration, *CC50* concentration of cytotoxicity 50%. *SI* = selectivity index of a drug is the ratio of the toxic concentration to its effective bioactive concentration = CC50/IC50 ≥ 10, non-toxic

Table 4 presents the IC50 values for leptin (Lep) and glucantime (Glu) alone and in combination (Lep + Glu) against promastigotes and amastigotes of *Leishmania tropica*. It also includes the cytotoxic values (CC50) of the drugs alone and combined on macrophages, allowing for the calculation of the selectivity index (SI).

### Impacts of leptin and glucantime and their combination on the production of ROS by the *Leishmania*-infected THP1 cells

**Fig. 2:**
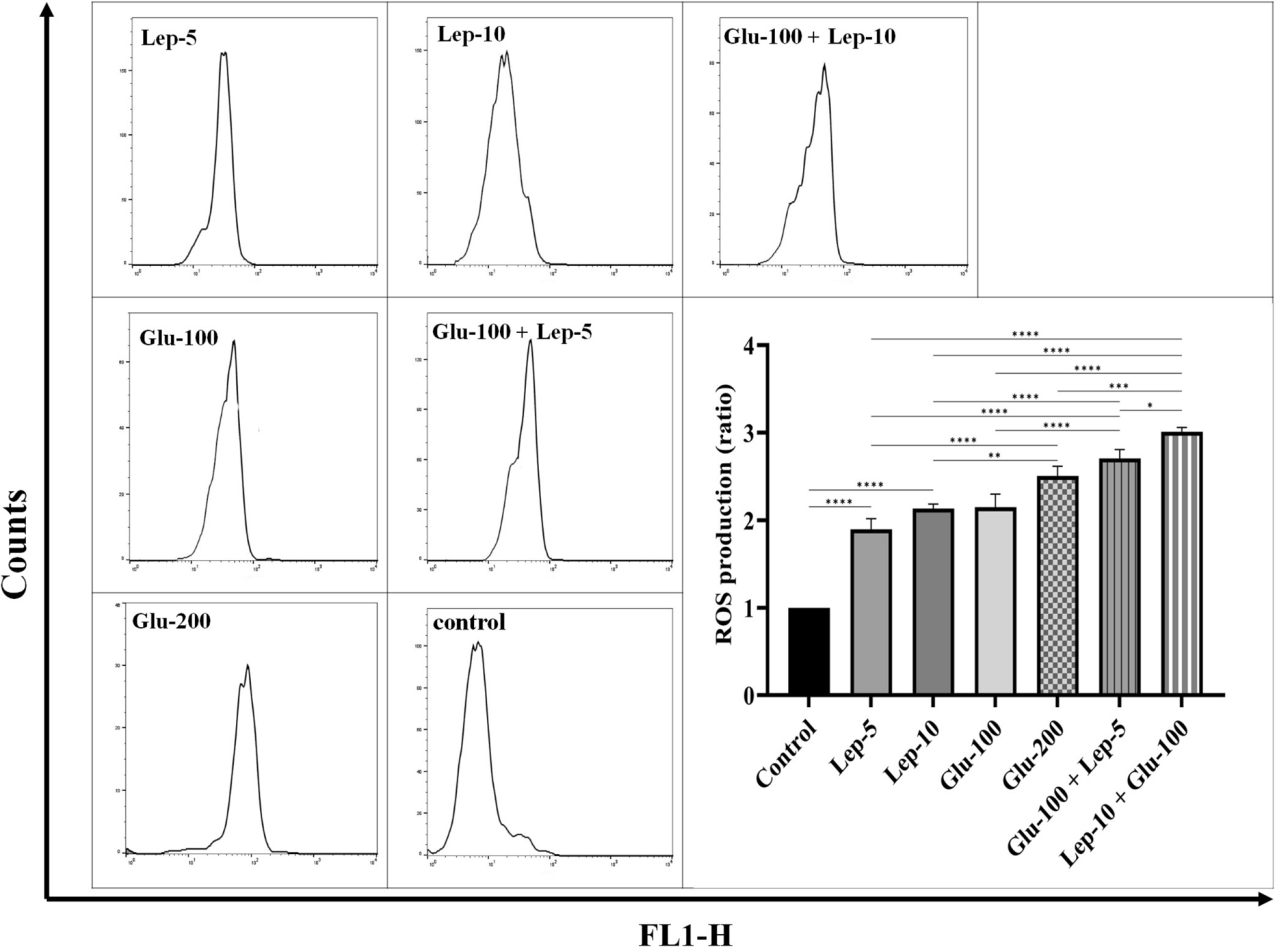
ROS production by *Leishmania*-infected THP1 cells in cultures treated with leptin, glucantime, and their combinations. Flowcytometry diagrams indicating the ROS production in samples treated with Lep-5, Lep-10, control, Glu-100 + Lep-5, Glu-100 + Lep-5, Glu-100, and Glu-200: To evaluate ROS production, *Leishmania tropica*-infected macrophages were treated with leptin, glucantime, or their combinations and incubated at 37 °C for 72 h. After incubation, the cells were washed and resuspended in PBS containing the cell-permeable probe CM-H2DCFDA. The suspension was then incubated at 37 °C for 30 min. Following another wash step, the fluorescence intensity of the cells was measured using flow cytometry, specifically in the FL1/FITC channel to quantify ROS generation by the macrophages. ROS levels in treated macrophages were calculated relative to those in untreated infected macrophages and expressed as a ratio. Experiments were conducted in triplicate, and results are presented as mean ± SD. Statistical analysis was performed using one-way ANOVA followed by the Student’s t-test to compare ROS production across cultures (*P < 0.05, **P < 0.01, ***P < 0.001, and ****P < 0.0001)

The ROS production by parasite-infected macrophages treated with Glu-100, Glu-200, Lep-5, Lep-10, Glu-100 + Lep-5, and Glu-100 + Lep-10 groups was significantly higher than that of the control group (all with *P* < 0.0001; Additional file 1: Table S1). ROS production by parasite-infected macrophages treated with Glu-200 and Lep-10, respectively, was significantly higher than that of those treated with Glu-100 and Lep-5 (*P* < 0.01 and *P* < 0.001, respectively). Additionally, ROS production by parasite-infected macrophages treated with the combinations Glu-100 + Lep-10 and Glu-100 + Lep-5 was significantly higher than that of those treated with Glu-200, Glu-100, Lep-10, and Lep-5 (Fig. 2 and Table S1). These results indicate that the combination of Glu-100 + Lep-10 or Glu-100 + Lep-5 were more effective than either monotherapy in terms of ROS production. Furthermore, infected macrophages treated with Glu-100 + Lep-10 produced greater amounts of ROS than those treated with Glu-100 + Lep-5 (*P* < 0.01; Fig. 2). More comparisons regarding ROS production between different control and treated macrophages are summarized in Table S1.

### The impacts of leptin and glucantime and their combinations on the expression of M1/M2-related markers in the *Leishmania*-infected THP1 cells.

#### Leptin, glucantime, or their combinations increased NOS2 expression and NO production while reducing Arg-1 expression

**Fig. 3:**
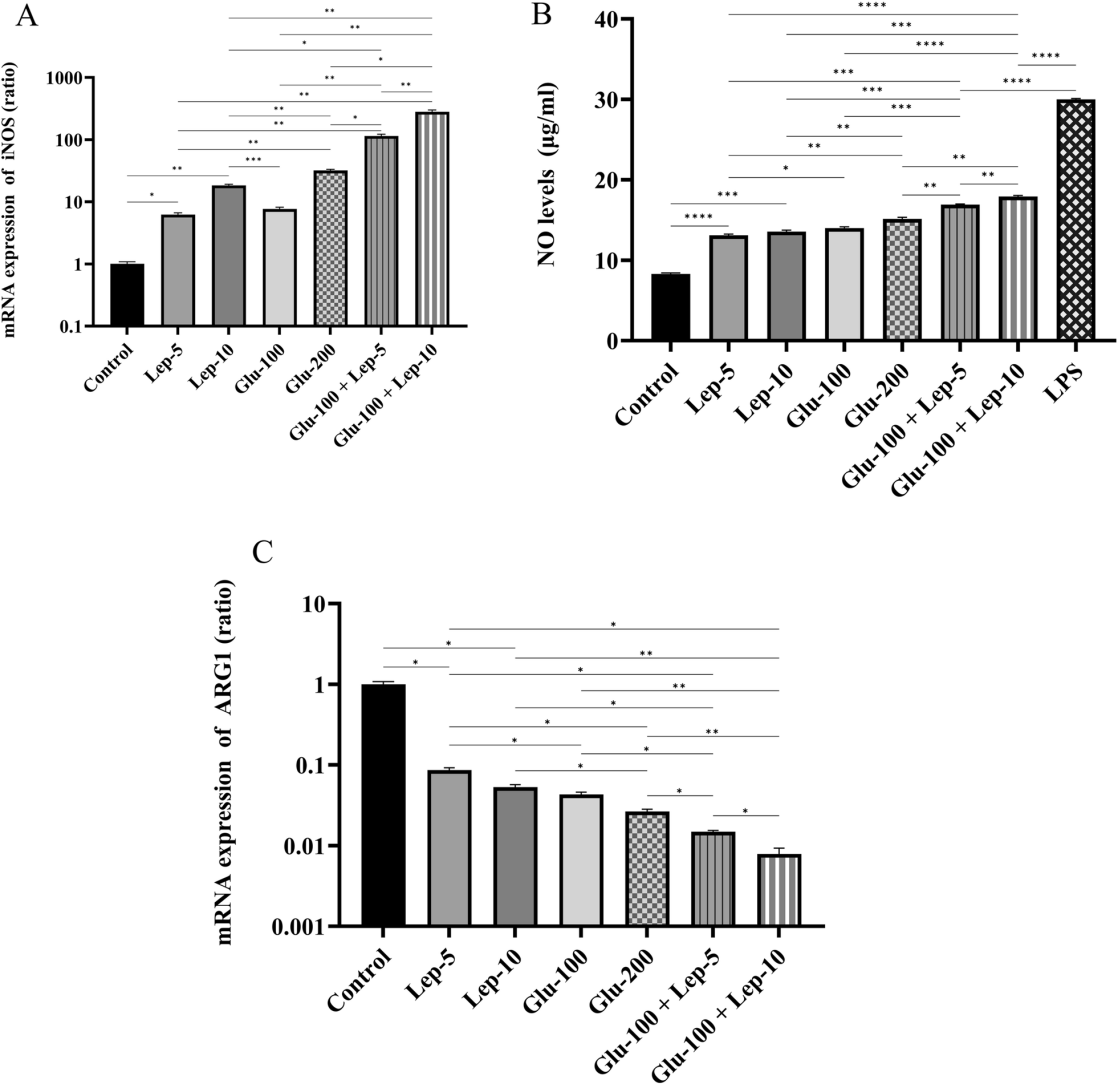
The NOS and ARG1 expression by *Leishmania*-infected THP1 cells treated with leptin, glucantime, and their combination. **A** mRNA expression of iNOS. **B** NO production. **C** mRNA expression of ARG1. To evaluate mRNA expression of iNOS and ARG-1 as well as NO production, macrophages infected with *Leishmania tropica* were treated with leptin, glucantime, or their combinations and incubated at 37 °C for 24 or 72 h. Untreated and LPS-stimulated macrophages served as negative and positive controls, respectively. Total RNA was extracted at 24 h and converted to cDNA. Real-time PCR was employed to measure the mRNA levels of iNOS and ARG-1. To assess NO production, cell supernatants were collected at 72 h and analyzed using the Griess test. All experiments were performed in triplicate, and results are expressed as mean ± SD. One-way ANOVA followed by Student’s t-test was used to compare variables between cultures (*P < 0.05, **P < 0.01, ***P < 0.001, and ****P < 0.0001)

The mRNA expression of iNOS in parasite-infected macrophages was significantly higher in groups treated with Glu-100, Glu-200, Lep-5, Lep-10, Glu-100 + Lep-5, and Glu-100 + Lep-10 compared to the control group (all with *P* < 0.05; Additional file 1: Table S2A). Among these, macrophages treated with Glu-200 and Lep-10, respectively, exhibited significantly higher iNOS mRNA expression than those treated with Glu-100 and Lep-5 (*P* < 0.001 and *P* < 0.001, respectively). Furthermore, the combination treatments of Glu-100 + Lep-10 and Glu-100 + Lep-5 resulted in notably higher iNOS mRNA expression than treatments with Glu-200, Glu-100, Lep-10, or Lep-5 alone (*P* < 0.01; Fig. 3A).

Similarly, NO production by parasite-infected macrophages treated with Glu-200 was significantly higher than for those treated with Glu-100 (*P* < 0.05). When treated with combinations of Glu-100 + Lep-10, and Glu-100 + Lep-5, NO production was significantly higher compared to treatments with Glu-200, Glu-100, Lep-10, or Lep-5 alone (*P* < 0.01, Fig. 3A). These findings suggest that the combination of Glu-100 + Lep-10 or Glu-100 + Lep-5 is more effective than monotherapy in enhancing iNOS expression and NO production. Furthermore, macrophages treated with Glu-100 + Lep-10 showed greater iNOS expression and NO production than those treated with Glu-100 + Lep-5 (Fig. 3A, B, and Additional file 1: Table S2A, B).

The mRNA expression of ARG1 by parasite-infected macrophages treated with Glu-100, Glu-200, Lep-5, Lep-10, Glu-100 + Lep-5, and Glu-100 + Lep-10 was significantly lower than that of untreated control macrophages (all with *P* < 0.05; Additional file 1: Table S2C). ARG1 mRNA expression by parasite-infected macrophages treated with Glu-200 and Lep-10, respectively, was significantly lower than that of those treated with Glu-100 and Lep-5 (*P* < 0.05 and *P* < 0.05, respectively). ARG1 mRNA expression by parasite-infected macrophages treated with combinations of Glu-100 + Lep-10 and Glu-100 + Lep-5 was significantly lower than for those treated with Glu-200, Glu-100, Lep-10, and Lep-5 alone (*P* < 0.05, Fig. 3C). These results also indicated that a combination of Glu-100 + Lep-10 or Glu-100 + Lep-5 was more effective than either monotherapy in reducing ARG1 expression. Furthermore, infected macrophages treated with Glu-100 + Lep-10 expressed lower levels of ARG1 than those treated with Glu-100 + Lep-5 (*P* < 0.05; Fig. 3C). More comparisons regarding NOS2, NO, and ARG1 expression between different control and treated macrophages are summarized in Table S2A, Table S2B and Table S2C, respectively.

#### Leptin, glucantime, or their combination modulated the expression of M1/M2-related cytokines by *Leishmania*-infected THP1 cells

**Fig. 4:**
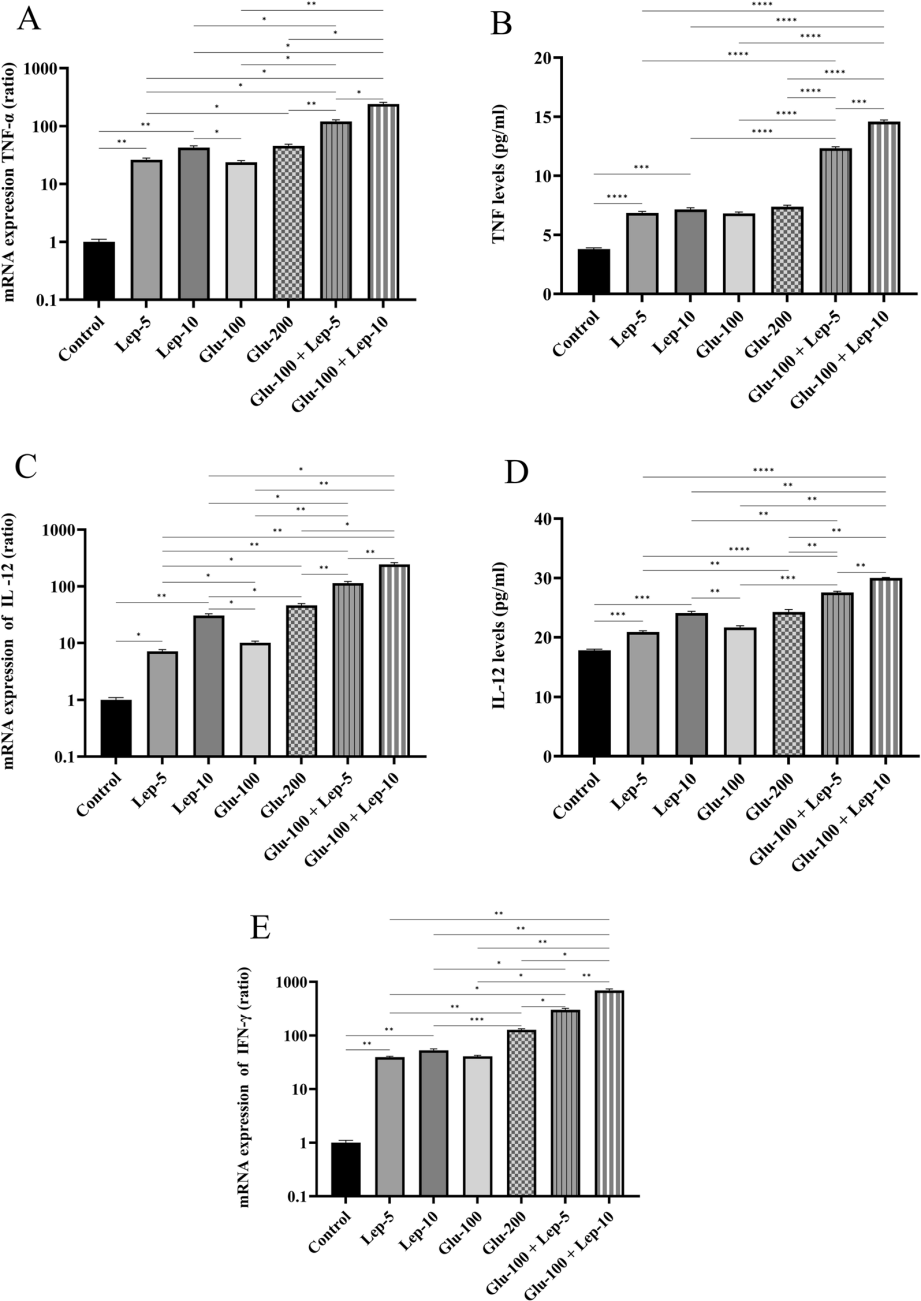
The TNF-α, IL-12, and IFN-γ expression of *Leishmania*-infected THP1 cells treated with leptin, glucantime, and their combination. **A** mRNA expression of TNF-α. **B** Protein expression of TNF-α. **C** mRNA expression of IL-12. **D** Protein expression of IL-12. **E** mRNA expression of IFN-γ. To assess mRNA and protein expression of M1-related cytokines, *Leishmania tropica*-infected macrophages were treated with leptin, glucantime, or their combinations and incubated at 37 °C for 24 or 72 h. The total RNA was extracted at 24 h and then converted to cDNA. Real-time PCR was used to determine the mRNA expression of TNF-α, IL-12, and IFN-γ. To determine TNF-α and IL-12 production, the cell supernatants were harvested at 72 h, and the cytokine amounts were assessed using the ELISA method. The experiments were performed in triplicate, and the results are expressed as mean ± SD. One-way ANOVA followed by Student's t-test was used to compare variables between cultures (*P < 0.05, **P < 0.01, ***P < 0.001, and ****P < 0.0001)

The protein and mRNA expression levels of TNF-α and IL-12 as well as mRNA expression of IFN-γ in parasite-infected macrophages treated with Glu-100, Glu-200, Lep-5, Lep-10, Glu-100 + Lep-5, and Glu-100 + Lep-10 were significantly higher compared to the control group (all with *P* < 0.05; Additional file 1: Table S3A, Table S3B, Table S3C, Table S3D and Table S3E). Notably, the mRNA expression of these M1-related cytokines was significantly elevated in macrophages treated with Glu-200 and Lep-10, respectively, compared to those treated with Glu-100 and Lep-5 (*P* < 0.05 and *P* < 0.05, respectively; Fig. 4). Additionally, the protein expression levels of TNF-α and IL-12 were significantly greater in macrophages treated with Glu-200 than in those treated with Glu-100 (*P* < 0.05, Fig. 4A and B). Moreover, IL-12 protein expression was significantly higher in macrophages treated with Lep-10 compared to those treated with Lep-5 (*P* < 0.01, Fig. 4C and D).

The protein and mRNA expression of TNF-α and IL-12, as well as the mRNA expression of IFN-γ, by parasite-infected macrophages was significantly higher when treated with the combinations Glu-100 + Lep-10 and Glu-100 + Lep-5 compared to treatments with Glu-200, Glu-100, Lep-10, and Lep-5 individually (*P* < 0.05, Fig. 4A–D). This suggests that the combination therapies Glu-100 + Lep-10 and Glu-100 + Lep-5 are more effective than monotherapies in enhancing TNF-α, IL-12, and IFN-γ expression. Moreover, macrophages treated with Glu-100 + Lep-10 exhibited higher levels of TNF-α, IL-12, and IFN-γ mRNA and protein than those treated with Glu-100 + Lep-5 (*P* < 0.05). Both Glu-200 and Glu-100 demonstrated a stronger capacity to induce TNF-α, IL-12, and IFN-γ expression compared to Lep-10 and Lep-5 (Additional file 1: Table S3A, Table S3B, Table S3C, Table S3D, and Table S3E).

**Fig. 5:**
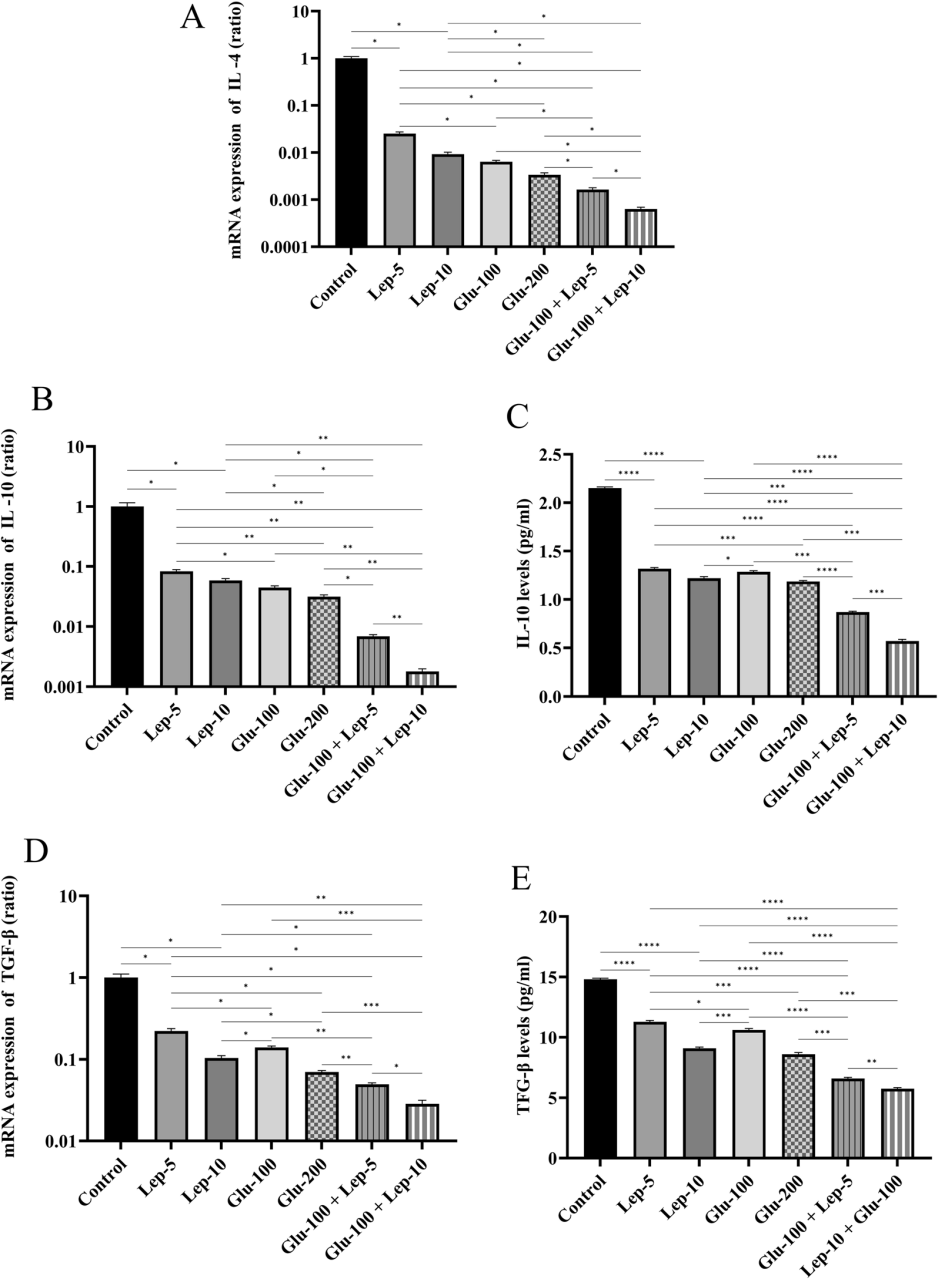
IL-4, IL-10, and TGF-β expression by *Leishmania*-infected THP1 cells treated with leptin, glucantime, and their combination. **A** mRNA expression of IL-4. **B** mRNA expression of IL-10. **C** Protein expression of IL-10. **D** mRNA expression of TGF-β. **E** Protein expression of TGF-β. To assess mRNA and protein expression of M2-related cytokines, *Leishmania tropica*-infected macrophages were treated with leptin, glucantime, or their combinations and incubated at 37 °C for 24 or 72 h. The total RNA was extracted at 24 h and then converted to cDNA. Real-time PCR was used to determine the mRNA expression of IL-4, IL-10, and TGF-β. To determine IL-10 and TGF-β production, the cell supernatants were harvested at 72 h, and cytokine amounts were assessed using the ELISA method. The experiments were performed in triplicate, and the results are expressed as mean ± SD. One-way ANOVA followed by Student's t-test was used to compare variables between cultures (*P < 0.05, **P < 0.01, ***P < 0.001, and ****P < 0.0001)

The protein and mRNA expression levels of IL-10 and TGF-β, as well as mRNA expression of IL-4, by parasite-infected macrophages treated with Glu-100, Glu-200, Lep-5, Lep-10, Glu-100 + Lep-5, and Glu-100 + Lep-10 were significantly reduced compared to untreated infected control macrophages (all with *P* < 0.05; Fig. 5A–E, Additional file 1: Table S4A, Table S4B, Table S4C, Table S4D, Table S4E). Additionally, the mRNA expression of IL-4, IL-10, and TGF-β in macrophages treated with Glu-200 and Lep-10, respectively, was lower than that in cells treated with Glu-100 and Lep-5 (*P* < 0.05). The protein and mRNA expression of IL-10 and TGF-β as well as mRNA expression of IL-4 by parasite-infected macrophages was significantly lower when treated with the combinations Glu-100 + Lep-10 and Glu-100 + Lep-5 compared to treatments with Glu-200, Glu-100, Lep-10, or Lep-5 alone (*P* < 0.05, Fig. 5B–E). These findings suggest that the combinations Glu-100 + Lep-10 or Glu-100 + Lep-5 are more effective than individual treatments in reducing IL-4, IL-10, and TGF-β expression. Additionally, macrophages treated with Glu-100 + Lep-10 showed  lower expression of IL-4, IL-10, and TGF-β than those treated with Glu-100 + Lep-5 (*P* < 0.05 and Fig. 5A–E).

In parasite-infected macrophages, the expression of IL-4, IL-10, and TGF-β was more overexpressed when treated with Lep-5 and Lep-10 compared to Glu-200 and Glu-100 treatments. Glu-100 treatment resulted in higher expression of IL-4, IL-10, and TGF-β than Glu-200. Additionally, IL-10 and TGF-β protein expression showed greater overexpression in macrophages treated with Lep-5 compared to Lep-10 (Figs. 5C and E). More comparisons regarding the expression of M1/M2-related cytokines between different control and treated macrophages are presented in Table S3A-Table S4E.

#### Leptin, glucantime, or their combination modulate the expression of M1- and M2-related markers by *Leishmania*-infected THP1 cells

**Fig. 6:**
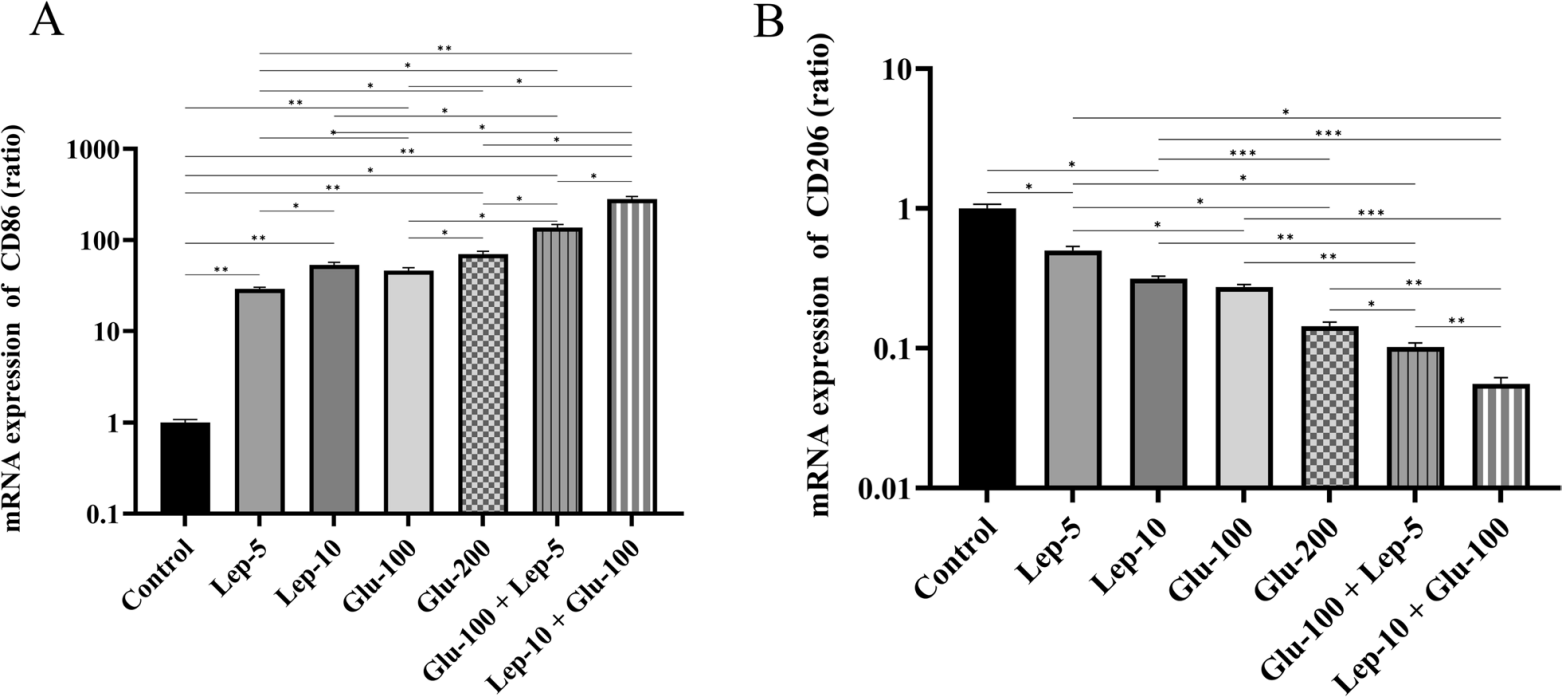
The CD86 and CD206 expression by *Leishmania*-infected THP1 cells treated with leptin, glucantime, and their combination. **A** mRNA expression of CD86. **B** mRNA expression of CD206. To assess mRNA expression of CD86 and CD206, *Leishmania tropica*-infected macrophages were treated with leptin, glucantime, or their combinations and incubated at 37 °C for 24 h. The total RNA was extracted at 24 h and then converted to cDNA. Real-time PCR was used to determine the mRNA expression of CD86 and CD206. The experiments were performed in triplicate, and the results are expressed as mean ± SD. One-way ANOVA followed by Student's t-test was used to compare variables between cultures (*P < 0.05, **P < 0.01, ***P < 0.001, and ****P < 0.0001)

The mRNA expression of CD86 by parasite-infected macrophages treated with Glu-100, Glu-200, Lep-5, Lep-10, Glu-100 + Lep-5, and Glu-100 + Lep-10 groups was significantly higher than that in the control group (all with *P* < 0.05; Fig. 6A, Additional file 1: Table S5A). The CD86 mRNA expression by parasite-infected macrophages treated with Glu-200 and Lep-10 was significantly higher than that of those treated with Glu-100 and Lep-5 (*P* < 0.01 and *P* < 0.05, respectively). CD86 mRNA expression by parasite-infected macrophages treated with combinations of Glu-100 + Lep-10 and Glu-100 + Lep-5 was significantly higher than for those treated with Glu-200, Glu-100, Lep-10, and Lep-5 alone (*P* < 0.05, Fig. 6A). These results indicate that a combination of Glu-100 + Lep-10 or Glu-100 + Lep-5 was more effective than either monotherapy regarding CD86 expression. In addition, infected macrophages treated with Glu-100 + Lep-10 expressed higher levels of CD86 than those treated with Glu-100 + Lep-5 (*P* < 0.05, Fig. 6A).

The mRNA expression of CD206 in parasite-infected macrophages was significantly reduced when treated with Glu-100, Glu-200, Lep-5, Lep-10, and combinations of Glu-100 + Lep-5 and Glu-100 + Lep-10 compared to untreated control macrophages (all with *P* < 0.05; Fig. 6B, Additional file 1: Table S5B). Treatment with Glu-200 and Lep-10 resulted in a greater reduction in CD206 expression than treatment with Glu-100 and Lep-5 (*P* < 0.01 and *P* < 0.05, respectively). Additionally, the combinations Glu-100 + Lep-10 and Glu-100 + Lep-5 were more effective in lowering CD206 expression than individual treatments with Glu-200, Glu-100, Lep-10, or Lep-5 (*P* < 0.05, Fig. 6B). Furthermore, macrophages treated with Glu-100 + Lep-10 exhibited lower CD206 levels than those treated with Glu-100 + Lep-5 (*P* < 0.01; Fig. 6B). More comparisons regarding the expression of CD86 and CD206 between different control and treated macrophages are presented in Table S5A and Table S5B.

#### Leptin, glucantime, or their combination modulates the SOCS3 and SOCS1 expression by *Leishmania*-infected THP1 cells

**Fig. 7:**
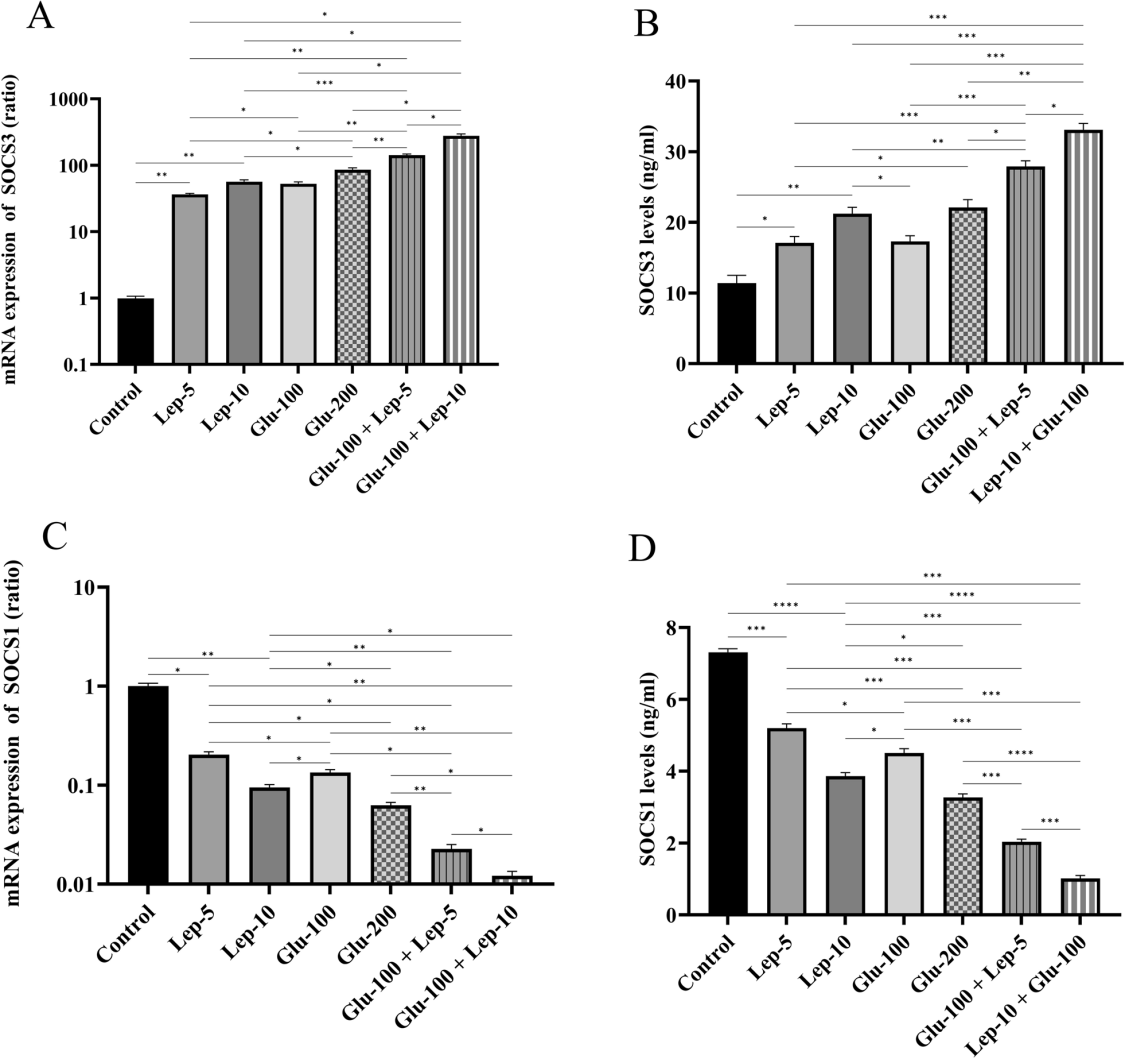
The SOCS expression by *Leishmania*-infected THP1 cells treated with leptin, glucantime, and their combination. **A** mRNA expression of SOCS3. **B** Protein expression of SOCS3. **C** mRNA expression of SOCS1. **D** protein expression of SOCS1. To assess mRNA and protein expression of SOCS molecules, *Leishmania tropica*-infected macrophages were treated with leptin, glucantime, or their combinations and incubated at 37 °C for 24 or 72 h. The total RNA was extracted at 24 h and then converted to cDNA. Real-time PCR was used to determine the mRNA expression of SOCS1 and SOCS3. To determine SOCS1 and SOCS3 production, the cell supernatants were harvested at 72 h, and the amounts of SOCS molecules were assessed using the ELISA method. The experiments were performed in triplicate, and the results are expressed as mean ± SD. One-way ANOVA followed by Student's t-test was used to compare variables between cultures (*P < 0.05, **P < 0.01, ***P < 0.001, and ****P < 0.0001)

The mRNA and protein expression of SOCS3 in parasite-infected macrophages was significantly elevated in groups treated with Glu-100, Glu-200, Lep-5, Lep-10, Glu-100 + Lep-5, and Glu-100 + Lep-10 compared to the control group (all with *P* < 0.01; Fig. 7A and B, Additional file 1: Table S6A and Table S6B). Among these, treatment with Glu-200 and Lep-10, respectively, resulted in higher SOCS3 mRNA expression than treatment with Glu-100 and Lep-5 (*P* < 0.05; Fig. 7A, Additional file 1: Table S6A). Furthermore, SOCS3 mRNA expression in macrophages treated with the combinations of Glu-100 + Lep-10 and Glu-100 + Lep-5 was significantly higher than in those treated with Glu-200, Glu-100, Lep-10, or Lep-5 alone (*P* < 0.05; Figs. 7A).

Similarly, SOCS3 production by parasite-infected macrophages treated with Glu-200 and Lep-10 was significantly higher than in those treated with Glu-100 and Lep-5 (*P* < 0.01; Table S6B). SOCS3 production by parasite-infected macrophages treated with combinations of Glu-100 + Lep-10 and Glu-100 + Lep-5 was significantly higher than that of those treated with Glu-200, Glu-100, Lep-10, and Lep-5 alone (*P* < 0.05; Fig. 7A and B). These results indicate that a combination of Glu-100 + Lep-10 or Glu-100 + Lep-5 was more effective than either monotherapy in terms of SOCS3 expression and production. In addition, infected macrophages treated with Glu-100 + Lep-10 produced higher levels of SOCS3 than those treated with Glu-100 + Lep-5 (*P* < 0.05; Figs. 7B).

The mRNA and protein expression of SOCS1 in parasite-infected macrophages treated with Glu-100, Glu-200, Lep-5, Lep-10, Glu-100 + Lep-5, or Glu-100 + Lep-10 was significantly lower than in the control group (all with *P* < 0.05; Fig. 7C and D, Additional file 1: Table S6C, Table S6D). SOCS1 mRNA expression in parasite-infected macrophages treated with Glu-200 and Lep-10 was significantly lower than in those treated with Glu-100 and Lep-5 (*P* < 0.05; Fig. 7C). SOCS1 mRNA expression was substantially lower in macrophages infected with parasites and treated with the combinations Glu-100 + Lep-10 or Glu-100 + Lep-5 than in those treated with Glu-200, Glu-100, Lep-10, or Lep-5 alone (*P* < 0.05; Fig. 7C and Table S6C). These results suggest that the combination of Glu-100 and Lep-10 or Glu-100 and Lep-5 is more effective than monotherapy in reducing SOCS1 expression. Furthermore, macrophages infected with the parasite and treated with Glu-100 + Lep-10 expressed lower amounts of SOCS1 than those treated with Glu-100 + Lep-5 (*P* < 0.05; Figs. 7C).

Similarly, the production of SOCS1 by parasite-infected macrophages was significantly lower when they were treated with Glu-200 and Lep-10 compared to treatments with Glu-100 and Lep-5 (*P* < 0.05 and *P* < 0.01, respectively). Additionally, macrophages treated with the combinations Glu-100 + Lep-10 and Glu-100 + Lep-5 showed a marked reduction in SOCS1 production compared to those treated individually with Glu-200, Glu-100, Lep-10, and Lep-5 alone (*P* < 0.001; Fig. 7D and Table S6D). These findings suggest that the combinations Glu-100 + Lep-10 or Glu-100 + Lep-5 are more effective than single-agent treatments in reducing SOCS1 protein and mRNA expression. Moreover, macrophages treated with Glu-100 + Lep-10 exhibited lower levels of SOCS1 protein and mRNA than those treated with Glu-100 + Lep-5 (*P* < 0.001; Fig. 7D).

#### Leptin, glucantime, or their combination modulated the expression of miR-155 and miR-146a by *Leishmania*-infected THP1 cells

**Fig. 8:**
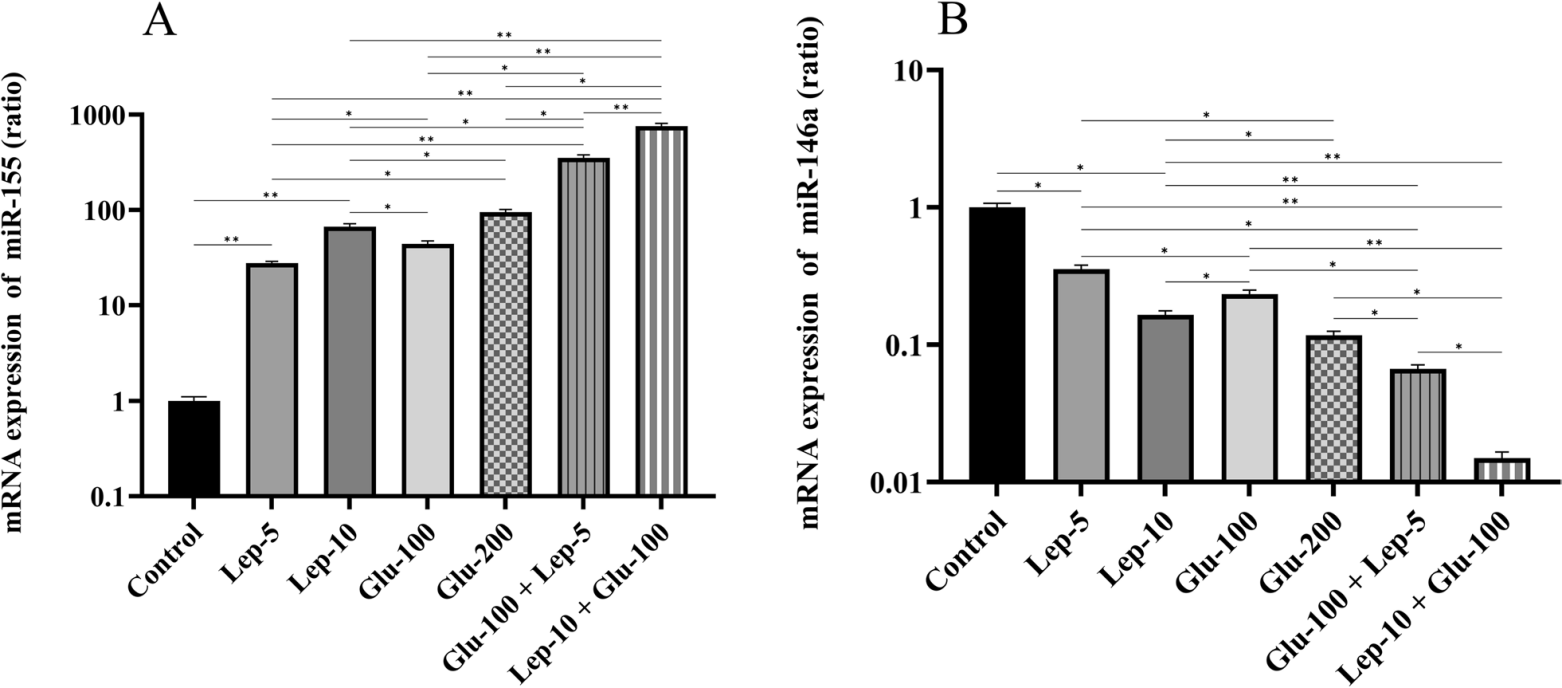
The miR-155 and miR-146a expression by *Leishmania*-infected THP1 cells treated with leptin, glucantime, and their combination. **A** miR-155 expression. **B** miR-146a expression. To assess mRNA expression of miR-155 and miR-146a, *Leishmania tropica*-infected macrophages were treated with leptin, glucantime, or their combinations and incubated at 37 °C for 24 h. The total RNA was extracted at 24 h and then converted to cDNA. Real-time PCR was used to determine the mRNA expression of miR-155 and miR-146a. The experiments were performed in triplicate, and the results are expressed as mean ± SD. One-way ANOVA followed by Student's t-test was used to compare variables between cultures (*P < 0.05, **P < 0.01, ***P < 0.001, and ****P < 0.0001)

The expression of the miR-155 by parasite-infected macrophages was significantly higher in the Glu-100, Glu-200, Lep-5, Lep-10, Glu-100 + Lep-5, and Glu-100 + Lep-10 groups than in the control group (all with *P* < 0.01; Fig. 8A, Additional file 1: Table S7A). The expression of miR-155 by parasite-infected macrophages treated with Glu-200 and Lep-10, respectively, was greater than that of the macrophages treated with Glu-100 and Lep-5 (*P* < 0.01 and *P* < 0.05, respectively). The expression of miR-155 by parasite-infected macrophages treated with the combination of Glu-100 and Lep-10 or Glu-100 and Lep-5 was substantially higher than that of the groups treated with Glu-200, Glu-100, Lep-10, or Lep-5 alone (*P* < 0.05; Fig. 8A and Table S7A). These results suggest that the combination of Glu-100 and Lep-10 or Glu-100 and Lep-5 is more effective than monotherapy in terms of miR-155 expression. Furthermore, macrophages infected with the parasite and treated with Glu-100 + Lep-10 expressed greater amounts of miR-155 than macrophages infected with the parasite and treated with Glu-100 + Lep-5 (*P* < 0.01; Fig. 8A).

The expression of miR-146a in parasite-infected macrophages treated with Glu-100, Glu-200, Lep-5, Lep-10, Glu-100 + Lep-5, or Glu-100 + Lep-10 was significantly reduced compared to untreated control macrophages (all with *P* < 0.05; Fig. 8B, Additional file 1: Table S7B). The miR-146a levels in macrophages treated with Glu-200 and Lep-10 were notably lower than those treated with Glu-100 and Lep-5 (*P* < 0.05 and *P* < 0.01, respectively). Additionally, macrophages treated with the combinations of Glu-100 and Lep-10 or Glu-100 and Lep-5 showed a significantly greater reduction in miR-146a expression than those treated with Glu-200, Glu-100, Lep-10, or Lep-5 alone (*P* < 0.05; Fig. 8B, Additional file 1: Table S7B). These findings suggest that combining Glu-100 with Lep-10 or Lep-5 is more effective than single treatments in decreasing miR-146a expression. Furthermore, macrophages treated with Glu-100 + Lep-10 exhibited lower miR-146a levels than those treated with Glu-100 + Lep-5 (*P* < 0.05; Fig. 8B).

#### Leptin, glucantime, and their combinations altered SOCS1 and SOCS3 expression in *Leishmania*-infected THP-1 cells

**Fig. 9:**
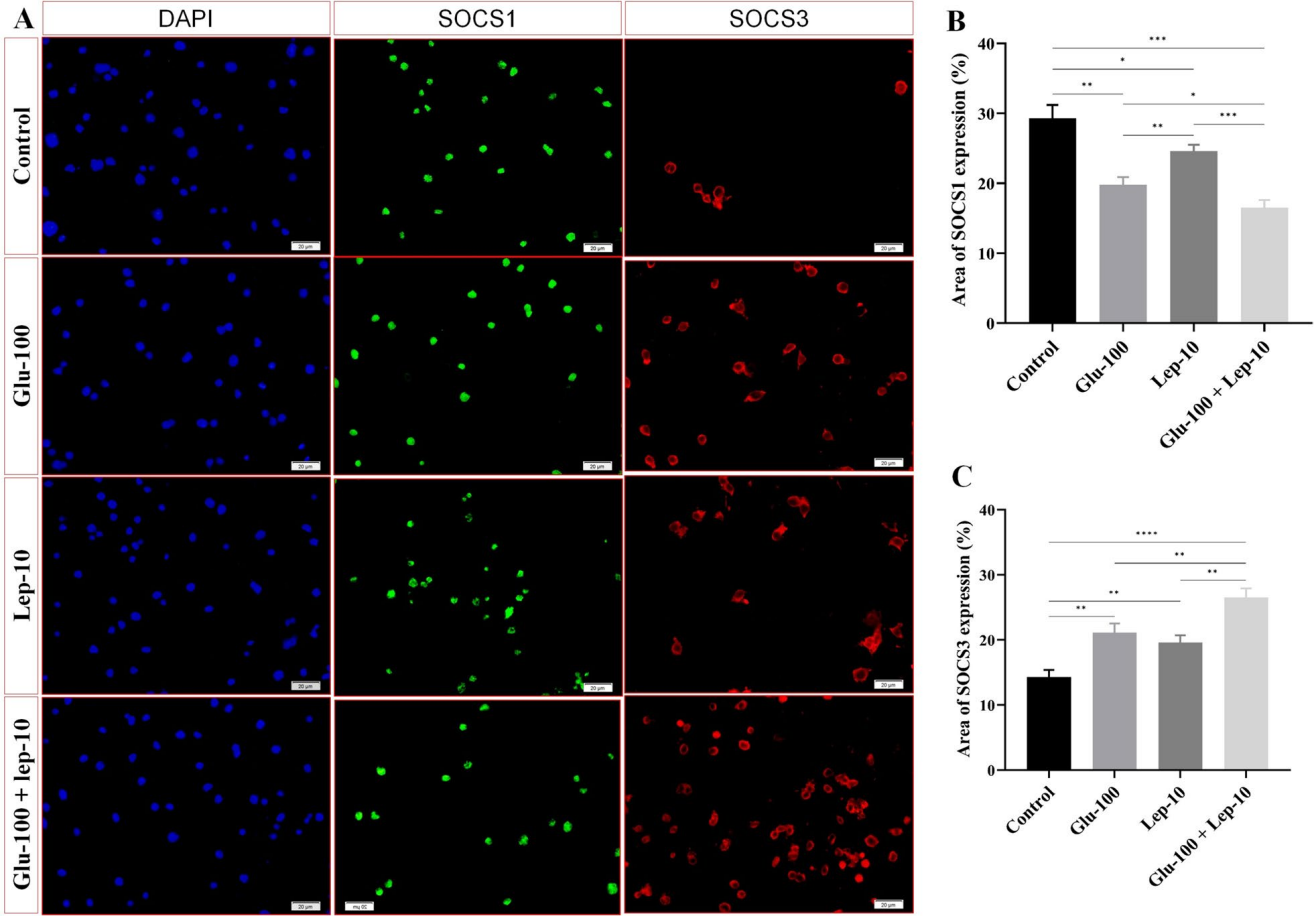
Immunofluorescence staining patterns of SOCS1 and SOCS3 expression in *Leishmania*-infected THP1 cells treated with leptin, glucantime, and their combination. **A** DAPI nuclear staining (blue), immunofluorescence green and red colors indicate SOCS1 and SOCS3 expression, respectively. Immunofluorescence staining illustrating the impact of leptin, glucantime, and their combination treatments on SOCS1 (FITC, green) and SOCS3 (CY3, red) protein expression in *Leishmania tropica*-infected macrophages after 72 h. Fluorescence intensity was analyzed via ImageJ, with results expressed as mean ± SD and statistically compared using one-way ANOVA and Student’s t-test (*P < 0.05, **P < 0.01, ***P < 0.001, and ****P < 0.0001)

Immunofluorescence analysis of *Leishmania tropica*-infected macrophages revealed contrasting levels of SOCS1 and SOCS3 proteins (Fig. 9). Specifically, the expression of SOCS1 by macrophages infected with the parasite and treated with Glu-100, Lep-10, or a combination of Glu-100 + Lep-10 was significantly lower than that of the untreated control macrophages (all with *P* < 0.05; Fig. 9A, Fig. 9B and Table S8A). SOCS1 expression by parasites infected with Glu-100 was significantly lower than that of parasites infected with Lep-10. Furthermore, the combination treatment resulted in a significantly greater reduction of SOCS1 than either monotherapy alone, including Glu-100 or Lep-10 (*P* < 0.05 and *P* < 0.001, respectively). The marked reduction in SOCS1 levels in the groups treated with leptin and glucantime, especially the combination therapy group, suggests a potential pathway for boosting immune responses by decreasing negative regulation.

Conversely, SOCS3 expression increased significantly in all treatment groups compared to the control group (all with *P* < 0.01; Fig. 9A, C and Table S8B). Notably, the combination treatment resulted in significantly higher SOCS3 expression than Glu-100 or Lep-10 alone (*P* < 0.01; Table S8B), suggesting a synergistic effect. These results indicate that the Glu-100 + Lep-10 combination is more effective than individual treatments in modulating SOCS1 and SOCS3 expression. A notable increase in SOCS3 protein levels across all treatment groups, particularly in the combination group, indicates a possible mechanism for boosting macrophage function in eliminating infection.

## Discussion

The M1 and M2 macrophages play crucial roles in resistance and susceptibility to *Leishmania* infection, respectively. The data of the present study demonstrated that both doses of leptin, alone and in combination with glucantime, shifted the balance of macrophage polarization toward the M1 phenotype, which is crucial for effective control of leishmanial infection. Leptin treatment upregulated M1-associated parameters (CD86, iNOS, SOCS3, miR-155) while downregulating M2-associated parameters (CD206, ARG1, SOCS1, miR-146a). These findings align with Li et al.’s observations that leptin enhances M1 polarization with increased expression of CD86, Nos2, and TNF-α [25]. Notably, Lep-10 demonstrated stronger efficacy compared to Lep-5 in promoting M1 polarization, as evidenced by the overexpression of M1-related markers and lower expression of M2-related markers. This dose-dependent effect suggests that the immunomodulatory potency of leptin can be mediated in a concentration-dependent manner.

Here, we observed that leptin-treated *L. tropica*-infected macrophages showed an increase in ROS and NO production. This is particularly important, as ROS and NO are vital effector molecules responsible for destroying parasites within macrophages [6]. Increased NO production, resulting from elevated iNOS expression, aligns with the M1 macrophage activation pathway. Both leptin concentrations (5 ng/ml and 10 ng/ml) elevated ROS levels, with combination treatments exhibiting synergistic effects. The dose-dependent increase in NO production (Lep-10 > Lep-5) and the enhanced efficacy of combination therapies suggest that leptin boosts the anti-parasitic capacity of infected macrophages in a concentration-dependent manner. In agreement with our findings, Raso et al. reported that leptin synergizes with inflammatory signals to enhance NO production by inducing iNOS expression [38]. NO exerts direct toxic effects on *Leishmania* parasites through various mechanisms, including inhibiting mitochondrial respiration, causing DNA damage, and disrupting iron-sulfur clusters in essential enzymes [7].

Similarly, the enhanced ROS production in leptin-treated infected macrophages aligns with Dayakar et al.’s observations that leptin enhances macrophage phagocytic activity by increasing intracellular ROS generation [12]. Elevated ROS production is crucial for parasite elimination, as it causes oxidative damage to parasite proteins, lipids, and nucleic acids [35]. The observed synergistic effect of leptin-glucantime combinations indicates that leptin not only directly stimulates ROS production but also amplifies the oxidative stress induced by glucantime, creating a highly hostile intracellular environment that impairs parasite survival.

Leptin treatment altered the cytokine profile of *L. tropica*-infected macrophages, promoting a pro-inflammatory environment conducive to parasite clearance. Both Lep-5 and Lep-10 increased the expression and generation of pro-inflammatory cytokines while reducing anti-inflammatory cytokines, with Lep-10 showing greater efficacy. This cytokine modulation pattern is consistent with Ahmed et al.’s findings that leptin increases monocyte/macrophage generation of pro-inflammatory cytokines [1] and Dayakar et al.’s observations of leptin-induced upregulation of pro-inflammatory cytokines in THP-1 cells [12].

The most significant finding of this study is the synergistic effect observed when leptin was combined with glucantime. Both combination treatments (Glu-100 + Lep-5 and Glu-100 + Lep-10) demonstrated substantially greater efficacy in all measured parameters compared to either agent alone. The combinations more potently upregulated M1 markers (CD86, SOCS3, miR-155) and downregulated M2 markers (CD206, SOCS1, miR-146a), enhanced ROS and NO production through increased iNOS expression and decreased ARG1 expression, and promoted the expression of pro-inflammatory cytokine expression while suppressing the expression anti-inflammatory cytokines.

The modulation of SOCS molecules by leptin treatment provides insights into the molecular mechanisms underlying leptin’s immunomodulatory effects. The immunofluorescence analysis of SOCS1 and SOCS3 protein expression provides essential mechanistic insights into leptin-mediated immunomodulatory effects at the protein level, complementing mRNA expression findings. The significant reduction in SOCS1 protein expression observed in treated macrophages has functional importance, as SOCS1 acts as a crucial negative regulator of pro-inflammatory signaling pathways, particularly the JAK-STAT pathway activated by IFN-γ and other Th1 cytokines [18].

Reduced SOCS1 expression following leptin treatment indicates that leptin alleviates a key inhibitory mechanism on pro-inflammatory responses, thereby enabling sustained and intensified M1 activation. This observation accounts for the overexpression of pro-inflammatory cytokines identified in our study. The combination treatments demonstrated an even stronger suppression of SOCS1, suggesting that leptin synergistically amplifies glucantime’s ability to sustain pro-inflammatory signaling. Interestingly, all treatments, especially the combination treatments, showed a marked upregulation of SOCS3 protein expression. SOCS3 specifically inhibits IL-10-mediated signaling pathways while exerting minimal effect on IFN-γ responses [14]. This selective upregulation of SOCS3 may serve as a mechanism through which leptin modulates the immune response, dampening anti-inflammatory IL-10 signaling while maintaining pro-inflammatory IFN-γ responses crucial for parasite clearance. Moreover, the upregulation of miR-155 (associated with M1 polarization) and downregulation of miR-146a (associated with M2 polarization) suggest that leptin influences macrophage polarization through multiple molecular pathways.

In agreement with our results, other studies showed that leptin reinforced LPS-induced M1 polarization of macrophages in vitro, demonstrating overexpression of TNF-α, NOS2, and CD86 gene as well as elevated protein expression of TNF-α, IL-6, NOS2 and CD86 [23, 25]. Moreover, Raso et al. demonstrated that leptin potently synergized with IFN-γ to enhance NO production in J774A.1 murine macrophages through increased expression of iNOS [38]. Ahmed et al. demonstrated that treatment of monocytes/macrophages with leptin increased the production of IL-1β and TNF-α and triggered the mRNA expression of caspase-1, which mediates the conversion of latent pro-IL-1β and pro-IL-18 to active forms [1]. Leptin also enhanced expression of IL-12, which contributes to T-lymphocyte proliferation [1].

Dayakar et al. examined leptin’s role in experimental VL, showing that leptin induced a Th1-specific response by upregulating pro-inflammatory cytokines in THP-1 cells and IFN-γ, IL-12, and IL-2 in peripheral blood mononuclear cells (PBMCs) [12]. They also revealed the reduced expression of M2 cytokine IL-10 in THP-1 cells. Leptin stimulated macrophages by triggering phosphorylation of Akt and Erk1/2, which are dephosphorylated during *L. donovani* infection. Furthermore, leptin enhanced phagocytosis efficacy of macrophages by increasing intracellular ROS generation, facilitating phagolysosome formation and ROS-mediated killing of the parasite [12].

Maurya et al. investigated the differential immunomodulatory role of leptin in controlling VL in normal and leptin-deficient mice [31]. Administration of the recombinant leptin diminished the splenic parasite burden in normal mice, correlating with enhanced innate immune responses, increased NO production, and elevated pro-inflammatory cytokines. Leptin treatment also reinforced the IFN-γ secretion from both CD4^+^ and CD8^+^ T cells. However, leptin-deficient mice showed higher parasite burden, and leptin treatment failed to reduce this burden or improve the cytokine response in these mice, suggesting differential effects based on baseline leptin status [31]. Fievez et al. found that diminished serum levels of leptin in VL patients were correlated positively with leukocyte counts and albumin and hemoglobin levels [11]. After 1 month of treatment, leptin levels significantly increased, reaching levels similar to those of healthy individuals. This suggests that leptin amounts are influenced during *Leishmania* infection and correlate with important prognostic parameters in VL [11].

The selectivity index data likely represent the most clinically significant findings of our study. The leptin-glucantime combination exhibited a higher selectivity index (SI) of 17.66 compared to individual treatments with leptin (7.88) or glucantime (6.87), highlighting its stronger antiparasitic efficacy. Glucantime exhibits both direct antiparasitic effects and immunomodulatory properties, while leptin primarily enhances immune responses [26, 29]. In agreement with our findings, it was reported that that leptin enhanced the efficacy of antileishmanial drugs, allowing for reduced drug concentrations [42]. Shivahare et al. investigated the immunomodulatory potential of leptin in combination with the standard anti-leishmanial drug, miltefosine. Leptin at 15 μg/ml enhanced Th1 cytokines and NO generation from murine macrophages, reducing the effective dose of miltefosine by twofold [42]. The combination of leptin and miltefosine increased the levels of IL-12, TNF-α, and NO while suppressing anti-inflammatory cytokines IL-10 and TGF-β in infected macrophages. This combination also enhanced the phagocytic ability of macrophages compared to either treatment alone. Combining leptin with reduced doses of miltefosine also significantly enhanced antileishmanial efficacy [42].

The synergistic effect of the leptin-glucantime combination is particularly promising from a therapeutic perspective. The combination of 10 ng/ml leptin and 100 μg/ml glucantime achieved complete elimination of intracellular amastigotes, marking a significant improvement over individual treatment. The ability to achieve enhanced antileishmanial effects with lower glucantime concentrations could potentially reduce treatment-associated toxicity while improving efficacy. This is supported by the selectivity index (SI) data, showing that the leptin-glucantime combination exhibited superior safety profiles compared to either agent alone.

This study has several limitations that should be considered. First, it used an in vitro model that may not fully replicate the complex immune environment of human leishmaniasis. Second, the research focused exclusively on one *Leishmania* species (*L. tropica*), and the results may not be generalizable to other species. Third, the long-term effects of leptin-glucantime combination therapy were not evaluated. Fourth, the potential development of leptin resistance during prolonged treatment was not investigated. In addition, the mechanisms by which leptin enhances the efficacy of glucantime require further investigation using in vivo models. Finally, additional studies are needed to determine the optimal dosing regimen for possible clinical use.

## Conclusions

This study demonstrates that leptin effectively modulates macrophage polarization and activation in *L. tropica*-infected macrophages, promoting an M1 phenotype conducive to parasite clearance. Leptin promotes M1 pro-inflammatory phenotype in macrophages, enhancing their leishmanicidal functions through increased production of ROS, NO, and pro-inflammatory cytokines. These effects contribute to an effective Th1 immune response crucial for controlling *Leishmania* infection. The ability of leptin to enhance the efficacy of conventional antileishmanial drugs at lower doses offers promising therapeutic potential. The synergistic effect observed when leptin is combined with glucantime offers promising therapeutic potential for leishmaniasis treatment, potentially allowing for reduced drug doses and associated toxicity while maintaining or enhancing efficacy. These findings provide a strong rationale for further investigation of leptin-based immunotherapeutic strategies in the management of leishmaniasis.

## Supplementary Information

Additional file 1.

## Data Availability

All data supporting this article’s conclusions can be found within the article itself.

## Abbreviations

SOCS1: Suppressor of cytokine signaling 1
SOCS3: Suppressor of cytokine signaling 3
MTT: 3-(4, 5-Dimethylthiazolyl-2)-2, 5-diphenyltetrazolium bromide
ROS: Reactive oxygen species
iNOS: Inducible nitric oxide synthase
NO: Nitric oxide
THP1: Tohoku Hospital Pediatrics-1 Cells
LPS: Lipopolysaccharide
IFN-γ: Interferon-gamma
TNF-α: Tumor necrosis factor-alpha
GM-CSF: Granulocyte-macrophage colony-stimulating factor
