# Supplementary material for: Leptin enhances the efficacy of glucantime to modulate macrophage polarization toward the M1 phenotype in Leishmania tropica-infected macrophages
Source: Parasit Vectors. 2025 Aug 25;18:360. doi: 10.1186/s13071-025-07004-6 (PMC12379411; doi:10.1186/s13071-025-07004-6)
Supplement: Supplementary file 1 — Additional file 1. [file 13071_2025_7004_MOESM1_ESM.docx]

**Additional file 1**

**Table S1: Statistical comparison of the ROS production by *leishmania*-infected THP1 cells treated with leptin and/or glucantime.**

| **Treatment types** | **Control** | **Glu-100** | **Glu-200** | **Lep-5** | **Lep-10** | **Glu-100 + Lep-5** | **Glu-100 + Lep-10** |
| --- | --- | --- | --- | --- | --- | --- | --- |
| **Control** | - | <0.0001 | <0.0001 | <0.0001 | <0.0001 | <0.0001 | <0.0001 |
| **Glu-100** | <0.0001 | - | 0.0066 | ns | ns | <0.0001 | <0.0001 |
| **Glu-200** | <0.0001 | 0.0066 | - | <0.0001 | 0.0047 | ns | 0.0002 |
| **Lep-5** | <0.0001 | ns | <0.0001 | - | ns | <0.0001 | <0.0001 |
| **Lep-10** | <0.0001 | ns | 0.0047 | ns | - | <0.0001 | <0.0001 |
| **Glu-100 + Lep-5** | <0.0001 | <0.0001 | ns | <0.0001 | <0.0001 | - | 0.0215 |
| **Glu-100 + Lep-10** | <0.0001 | <0.0001 | 0.0002 | <0.0001 | <0.0001 | 0.0215 | - |

**Table S2A: Statistical comparison of the mRNA expression of NOS2 by *leishmania*-infected THP1 cells treated with leptin and/or glucantime.**

| **Treatment types** | **Control** | **Glu-100** | **Glu-200** | **Lep-5** | **Lep-10** | **Glu-100 + Lep-5** | **Glu-100 + Lep-10** |
| --- | --- | --- | --- | --- | --- | --- | --- |
| **Control** | - | 0.0108 | 0.0028 | 0.0117 | 0.0029 | 0.0081 | 0.008 |
| **Glu-100** | 0.0108 | - | 0.0006 | ns | 0.0003 | 0.0092 | 0.0084 |
| **Glu-200** | 0.0028 | 0.0006 | - | 0.0045 | 0.0038 | 0.0157 | 0.0102 |
| **Lep-5** | 0.0117 | ns | 0.0045 | - | 0.0011 | 0.0089 | 0.0083 |
| **Lep-10** | 0.0029 | 0.0003 | 0.0038 | 0.0011 | - | 0.0114 | 0.0091 |
| **Glu-100 + Lep-5** | 0.0081 | 0.0092 | 0.0157 | 0.0089 | 0.0114 | - | 0.0062 |
| **Glu-100 + Lep-10** | 0.0080 | 0.0084 | 0.0102 | 0.0083 | 0.0091 | 0.0062 | - |

**Table S2B: Statistical comparison of the protein expression of NO by *leishmania*-infected THP1 cells treated with leptin and/or glucantime.**

| **Treatment types** | **Control** | **Glu-100** | **Glu-200** | **Lep-5** | **Lep-10** | **Glu-100 + Lep-5** | **Glu-100 + Lep-10** |
| --- | --- | --- | --- | --- | --- | --- | --- |
| **Control** | - | <0.0001 | 0.0001 | <0.0001 | 0.0002 | <0.0001 | <0.0001 |
| **Glu-100** | <0.0001 | - | 0.0322 | 0.0132 | ns | 0.0006 | <0.0001 |
| **Glu-200** | 0.0001 | 0.0322 | - | 0.0059 | 0.0056 | 0.0066 | 0.0019 |
| **Lep-5** | <0.0001 | 0.0132 | 0.0059 | - | ns | 0.0003 | <0.0001 |
| **Lep-10** | 0.0002 | ns | 0.0056 | ns | - | 0.0007 | 0.0004 |
| **Glu-100 + Lep-5** | <0.0001 | 0.0006 | 0.0066 | 0.0003 | 0.0007 | - | 0.0024 |
| **Glu-100 + Lep-10** | <0.0001 | <0.0001 | 0.0019 | <0.0001 | 0.0004 | 0.0024 | - |

**Table S2C: Statistical comparison of the mRNA expression of ARG1 by *leishmania*-infected THP1 cells treated with leptin and/or glucantime.**

| **Treatment types** | **Control** | **Glu-100** | **Glu-200** | **Lep-5** | **Lep-10** | **Glu-100 + Lep-5** | **Glu-100 + Lep-10** |
| --- | --- | --- | --- | --- | --- | --- | --- |
| **Control** | - | 0.011 | 0.0107 | 0.0122 | 0.0113 | 0.0104 | 0.0103 |
| **Glu-100** | 0.011 | - | 0.0273 | 0.0114 | ns | 0.0192 | 0.0026 |
| **Glu-200** | 0.0107 | 0.0273 | - | 0.0181 | 0.0112 | 0.0424 | 0.0014 |
| **Lep-5** | 0.0122 | 0.0114 | 0.0181 | - | 0.028 | 0.0118 | 0.0102 |
| **Lep-10** | 0.0113 | ns | 0.0112 | 0.028 | - | 0.0158 | 0.0021 |
| **Glu-100 + Lep-5** | 0.0104 | 0.0192 | 0.0424 | 0.0118 | 0.0158 | - | 0.0304 |
| **Glu-100 + Lep-10** | 0.0103 | 0.0026 | 0.0014 | 0.0102 | 0.0021 | 0.0304 | - |

**Table S3A: Statistical comparison of mRNA expression of the TNF-α by *leishmania*-infected THP1 cells treated with leptin and/or glucantime.**

| **Treatment types** | **Control** | **Glu-100** | **Glu-200** | **Lep-5** | **Lep-10** | **Glu-100 + Lep-5** | **Glu-100 + Lep-10** |
| --- | --- | --- | --- | --- | --- | --- | --- |
| **Control** | - | 0.0087 | 0.0083 | 0.0086 | 0.0083 | 0.0081 | 0.008 |
| **Glu-100** | 0.0087 | - | 0.013 | ns | 0.0172 | 0.0128 | 0.0099 |
| **Glu-200** | 0.0083 | 0.013 | - | 0.0197 | ns | 0.0053 | 0.0125 |
| **Lep-5** | 0.0086 | ns | 0.0197 | - | 0.0276 | 0.0136 | 0.0101 |
| **Lep-10** | 0.0083 | 0.0172 | ns | 0.0276 | - | 0.0213 | 0.0121 |
| **Glu-100 + Lep-5** | 0.0081 | 0.0128 | 0.0053 | 0.0136 | 0.0213 | - | 0.0113 |
| **Glu-100 + Lep-10** | 0.008 | 0.0099 | 0.0125 | 0.0101 | 0.0121 | 0.0113 | - |

**Table S3B: Statistical comparison of protein expression of the TNF-α by *leishmania*-infected THP1 cells treated with leptin and/or glucantime.**

| **Treatment types** | **Control** | **Glu-100** | **Glu-200** | **Lep-5** | **Lep-10** | **Glu-100 + Lep-5** | **Glu-100 + Lep-10** |
| --- | --- | --- | --- | --- | --- | --- | --- |
| **Control** | - | <0.0001 | <0.0001 | <0.0001 | 0.0005 | <0.0001 | <0.0001 |
| **Glu-100** | <0.0001 | - | 0.0385 | ns | ns | <0.0001 | <0.0001 |
| **Glu-200** | <0.0001 | 0.0385 | - | ns | ns | <0.0001 | <0.0001 |
| **Lep-5** | <0.0001 | ns | ns | - | ns | <0.0001 | <0.0001 |
| **Lep-10** | 0.0005 | ns | ns | ns | - | <0.0001 | <0.0001 |
| **Glu-100 + Lep-5** | <0.0001 | <0.0001 | <0.0001 | <0.0001 | <0.0001 | - | 0.0003 |
| **Glu-100 + Lep-10** | <0.0001 | <0.0001 | <0.0001 | <0.0001 | <0.0001 | 0.0003 | - |

**Table S3C: Statistical comparison of mRNA expression of the IL-12 by *leishmania*-infected THP1 cells treated with leptin and/or glucantime.**

| **Treatment types** | **Control** | **Glu-100** | **Glu-200** | **Lep-5** | **Lep-10** | **Glu-100 + Lep-5** | **Glu-100 + Lep-10** |
| --- | --- | --- | --- | --- | --- | --- | --- |
| **Control** | - | 0.0101 | 0.0083 | 0.0113 | 0.0085 | 0.0081 | 0.008 |
| **Glu-100** | 0.0101 | - | 0.0136 | 0.0365 | 0.0195 | 0.0096 | 0.0087 |
| **Glu-200** | 0.0083 | 0.0136 | - | 0.0114 | 0.0412 | 0.0062 | 0.0125 |
| **Lep-5** | 0.0113 | 0.0365 | 0.0114 | - | 0.0142 | 0.0091 | 0.0084 |
| **Lep-10** | 0.0085 | 0.0195 | 0.0412 | 0.0142 | - | 0.0159 | 0.0105 |
| **Glu-100 + Lep-5** | 0.0081 | 0.0096 | 0.0062 | 0.0091 | 0.0159 | - | 0.009 |
| **Glu-100 + Lep-10** | 0.008 | 0.0087 | 0.0125 | 0.0084 | 0.0105 | 0.009 | - |

**Table S3D: Statistical comparison of protein expression of the IL-12 by *leishmania*-infected THP1 cells treated with leptin and/or glucantime.**

| **Treatment types** | **Control** | **Glu-100** | **Glu-200** | **Lep-5** | **Lep-10** | **Glu-100 + Lep-5** | **Glu-100 + Lep-10** |
| --- | --- | --- | --- | --- | --- | --- | --- |
| **Control** | - | 0.0026 | 0.001 | 0.0005 | 0.0006 | <0.0001 | <0.0001 |
| **Glu-100** | 0.0026 | - | 0.0079 | ns | 0.0056 | 0.0007 | 0.0024 |
| **Glu-200** | 0.001 | 0.0079 | - | 0.0071 | ns | 0.0077 | 0.0086 |
| **Lep-5** | 0.0005 | ns | 0.0071 | - | 0.0045 | <0.0001 | <0.0001 |
| **Lep-10** | 0.0006 | 0.0056 | ns | 0.0045 | - | 0.0035 | 0.0048 |
| **Glu-100 + Lep-5** | <0.0001 | 0.0007 | 0.0077 | <0.0001 | 0.0035 | - | 0.0024 |
| **Glu-100 + Lep-10** | <0.0001 | 0.0024 | 0.0086 | <0.0001 | 0.0048 | 0.0024 | - |

**Table S3E: Statistical comparison of mRNA expression of the IFN-γ by *leishmania*-infected THP1 cells treated with leptin and/or glucantime.**

| **Treatment types** | **Control** | **Glu-100** | **Glu-200** | **Lep-5** | **Lep-10** | **Glu-100 + Lep-5** | **Glu-100 + Lep-10** |
| --- | --- | --- | --- | --- | --- | --- | --- |
| **Control** | - | 0.0029 | 0.0026 | 0.0028 | 0.0083 | 0.008 | 0.008 |
| **Glu-100** | 0.0029 | - | 0.0062 | ns | ns | 0.0107 | 0.009 |
| **Glu-200** | 0.0026 | 0.0062 | - | 0.0059 | 0.0003 | 0.0252 | 0.0121 |
| **Lep-5** | 0.0028 | ns | 0.0059 | - | ns | 0.0106 | 0.009 |
| **Lep-10** | 0.0083 | ns | 0.0003 | ns | - | 0.012 | 0.0094 |
| **Glu-100 + Lep-5** | 0.008 | 0.0107 | 0.0252 | 0.0106 | 0.012 | - | 0.0074 |
| **Glu-100 + Lep-10** | 0.008 | 0.009 | 0.0121 | 0.009 | 0.0094 | 0.0074 | - |

**Table S4A: Statistical comparison of mRNA expression of the IL-4 by *leishmania*-infected THP1 cells treated with leptin and/or glucantime.**

| **Treatment types** | **Control** | **Glu-100** | **Glu-200** | **Lep-5** | **Lep-10** | **Glu-100 + Lep-5** | **Glu-100 + Lep-10** |
| --- | --- | --- | --- | --- | --- | --- | --- |
| **Control** | - | 0.0139 | 0.0138 | 0.0144 | 0.014 | 0.0137 | 0.0137 |
| **Glu-100** | 0.0139 | - | 0.0078 | 0.0205 | ns | 0.0167 | 0.0104 |
| **Glu-200** | 0.0138 | 0.0078 | - | 0.015 | 0.015 | 0.0312 | 0.0278 |
| **Lep-5** | 0.0144 | 0.0205 | 0.015 | - | 0.0087 | 0.0126 | 0.0116 |
| **Lep-10** | 0.014 | ns | 0.015 | 0.0087 | - | 0.0258 | 0.0198 |
| **Glu-100 + Lep-5** | 0.0137 | 0.0167 | 0.0312 | 0.0126 | 0.0258 | - | 0.0131 |
| **Glu-100 + Lep-10** | 0.0137 | 0.0104 | 0.0278 | 0.0116 | 0.0198 | 0.0131 | - |

**Table S4B: Statistical comparison of mRNA expression of the IL-10 by *leishmania*-infected THP1 cells treated with leptin and/or glucantime.**

| **Treatment types** | **Control** | **Glu-100** | **Glu-200** | **Lep-5** | **Lep-10** | **Glu-100 + Lep-5** | **Glu-100 + Lep-10** |
| --- | --- | --- | --- | --- | --- | --- | --- |
| **Control** | - | 0.0407 | 0.0398 | 0.0441 | 0.0419 | 0.0377 | 0.0374 |
| **Glu-100** | 0.0407 | - | 0.0363 | 0.0146 | ns | 0.0113 | 0.0087 |
| **Glu-200** | 0.0398 | 0.0363 | - | 0.0061 | 0.0197 | 0.013 | 0.0089 |
| **Lep-5** | 0.0441 | 0.0146 | 0.0061 | - | 0.0363 | 0.0095 | 0.0083 |
| **Lep-10** | 0.0419 | ns | 0.0197 | 0.0363 | - | 0.0103 | 0.0085 |
| **Glu-100 + Lep-5** | 0.0377 | 0.0113 | 0.013 | 0.0095 | 0.0103 | - | 0.0032 |
| **Glu-100 + Lep-10** | 0.0374 | 0.0087 | 0.0089 | 0.0083 | 0.0085 | 0.0032 | - |

**Table S4C: Statistical comparison of protein expression of the IL-10 by *leishmania*-infected THP1 cells treated with leptin and/or glucantime.**

| **Treatment types** | **Control** | **Glu-100** | **Glu-200** | **Lep-5** | **Lep-10** | **Glu-100 + Lep-5** | **Glu-100 + Lep-10** |
| --- | --- | --- | --- | --- | --- | --- | --- |
| **Control** | - | <0.0001 | <0.0001 | <0.0001 | <0.0001 | <0.0001 | <0.0001 |
| **Glu-100** | <0.0001 | - | 0.0033 | ns | 0.0486 | 0.0001 | <0.0001 |
| **Glu-200** | <0.0001 | 0.0033 | - | 0.0009 | ns | <0.0001 | 0.0001 |
| **Lep-5** | <0.0001 | ns | 0.0009 | - | 0.0251 | <0.0001 | <0.0001 |
| **Lep-10** | <0.0001 | 0.0486 | ns | 0.0251 | - | 0.0005 | <0.0001 |
| **Glu-100 + Lep-5** | <0.0001 | 0.0001 | <0.0001 | <0.0001 | 0.0005 | - | 0.0009 |
| **Glu-100 + Lep-10** | <0.0001 | <0.0001 | 0.0001 | <0.0001 | <0.0001 | 0.0009 | - |

**Table S4D: Statistical comparison of mRNA expression of the TGF-β by *leishmania*-infected THP1 cells treated with leptin and/or glucantime.**

| **Treatment types** | **Control** | **Glu-100** | **Glu-200** | **Lep-5** | **Lep-10** | **Glu-100 + Lep-5** | **Glu-100 + Lep-10** |
| --- | --- | --- | --- | --- | --- | --- | --- |
| **Control** | - | 0.0241 | 0.0206 | 0.0299 | 0.0222 | 0.0197 | 0.0189 |
| **Glu-100** | 0.0241 | - | 0.0023 | 0.0232 | 0.0223 | 0.0073 | 0.0006 |
| **Glu-200** | 0.0206 | 0.0023 | - | 0.0174 | 0.0344 | 0.005 | 0.0007 |
| **Lep-5** | 0.0299 | 0.0232 | 0.0174 | - | 0.009 | 0.0133 | 0.0109 |
| **Lep-10** | 0.0222 | 0.0223 | 0.0344 | 0.009 | - | 0.0309 | 0.0035 |
| **Glu-100 + Lep-5** | 0.0197 | 0.0073 | 0.005 | 0.0133 | 0.0309 | - | 0.0159 |
| **Glu-100 + Lep-10** | 0.0189 | 0.0006 | 0.0007 | 0.0109 | 0.0035 | 0.0159 | - |

**Table S4E: Statistical comparison of protein expression of the TGF-β by *leishmania*-infected THP1 cells treated with leptin and/or glucantime.**

| **Treatment types** | **Control** | **Glu-100** | **Glu-200** | **Lep-5** | **Lep-10** | **Glu-100 + Lep-5** | **Glu-100 + Lep-10** |
| --- | --- | --- | --- | --- | --- | --- | --- |
| **Control** | - | <0.0001 | <0.0001 | <0.0001 | <0.0001 | <0.0001 | <0.0001 |
| **Glu-100** | <0.0001 | - | 0.0005 | 0.0222 | 0.0007 | <0.0001 | <0.0001 |
| **Glu-200** | <0.0001 | 0.0005 | - | 0.0001 | ns | 0.0003 | 0.0006 |
| **Lep-5** | <0.0001 | 0.0222 | 0.0001 | - | 0.0002 | <0.0001 | <0.0001 |
| **Lep-10** | <0.0001 | 0.0007 | ns | 0.0002 | - | <0.0001 | <0.0001 |
| **Glu-100 + Lep-5** | <0.0001 | <0.0001 | 0.0003 | <0.0001 | <0.0001 | - | 0.004 |
| **Glu-100 + Lep-10** | <0.0001 | <0.0001 | 0.0006 | <0.0001 | <0.0001 | 0.004 | - |

**Table S5A: Statistical comparison of mRNA expression of the CD86 by *leishmania*-infected THP1 cells treated with leptin and/or glucantime.**

| **Treatment types** | **Control** | **Glu-100** | **Glu-200** | **Lep-5** | **Lep-10** | **Glu-100 + Lep-5** | **Glu-100 + Lep-10** |
| --- | --- | --- | --- | --- | --- | --- | --- |
| **Control** | - | 0.0083 | 0.0082 | 0.0029 | 0.0083 | 0.0112 | 0.008 |
| **Glu-100** | 0.0083 | - | 0.0412 | 0.0232 | ns | 0.027 | 0.0117 |
| **Glu-200** | 0.0082 | 0.0412 | - | 0.0244 | ns | 0.0181 | 0.015 |
| **Lep-5** | 0.0029 | 0.0232 | 0.0244 | - | 0.042 | 0.018 | 0.0099 |
| **Lep-10** | 0.0083 | ns | ns | 0.042 | - | 0.0323 | 0.0125 |
| **Glu-100 + Lep-5** | 0.0112 | 0.027 | 0.0181 | 0.018 | 0.0323 | - | 0.0116 |
| **Glu-100 + Lep-10** | 0.008 | 0.0117 | 0.015 | 0.0099 | 0.0125 | 0.0116 | - |

**Table S5B: Statistical comparison of mRNA expression of the CD206 by *leishmania*-infected THP1 cells treated with leptin and/or glucantime.**

| **Treatment types** | **Control** | **Glu-100** | **Glu-200** | **Lep-5** | **Lep-10** | **Glu-100 + Lep-5** | **Glu-100 + Lep-10** |
| --- | --- | --- | --- | --- | --- | --- | --- |
| **Control** | **-** | 0.0154 | 0.0111 | 0.0113 | 0.0174 | 0.0099 | 0.009 |
| **Glu-100** | 0.0154 | **-** | 0.0011 | 0.042 | 0.1189 | 0.0014 | 0.0006 |
| **Glu-200** | 0.0111 | 0.0011 | **-** | 0.0169 | 0.0005 | 0.0362 | 0.0071 |
| **Lep-5** | 0.0113 | 0.042 | 0.0169 | **-** | 0.0232 | 0.013 | 0.0104 |
| **Lep-10** | 0.0174 | ns | 0.0005 | 0.0232 | **-** | 0.001 | 0.0005 |
| **Glu-100 + Lep-5** | 0.0099 | 0.0014 | 0.0362 | 0.013 | 0.001 | **-** | 0.0098 |
| **Glu-100 + Lep-10** | 0.009 | 0.0006 | 0.0071 | 0.0104 | 0.0005 | 0.0098 | **-** |

**Table S6A: Statistical comparison of mRNA expression of the SOCS3 by *leishmania*-infected THP1 cells treated with leptin and/or glucantime.**

| **Treatment types** | **Control** | **Glu-100** | **Glu-200** | **Lep-5** | **Lep-10** | **Glu-100 + Lep-5** | **Glu-100 + Lep-10** |
| --- | --- | --- | --- | --- | --- | --- | --- |
| **Control** | - | 0.0083 | 0.0081 | 0.0028 | 0.0083 | 0.0028 | 0.008 |
| **Glu-100** | 0.0083 | - | 0.0276 | 0.0395 | ns | 0.0014 | 0.0125 |
| **Glu-200** | 0.0081 | 0.0276 | - | 0.0252 | 0.0411 | 0.0028 | 0.0181 |
| **Lep-5** | 0.0028 | 0.0395 | 0.0252 | - | 0.0261 | 0.0052 | 0.0106 |
| **Lep-10** | 0.0083 | ns | 0.0411 | 0.0261 | - | 0.0003 | 0.013 |
| **Glu-100 + Lep-5** | 0.0028 | 0.0014 | 0.0028 | 0.0052 | 0.0003 | - | 0.0357 |
| **Glu-100 + Lep-10** | 0.008 | 0.0125 | 0.0181 | 0.0106 | 0.013 | 0.0357 | - |

**Table S6B: Statistical comparison of protein expression of the SOCS3 by *leishmania*-infected THP1 cells treated with leptin and/or glucantime.**

| **Treatment types** | **Control** | **Glu-100** | **Glu-200** | **Lep-5** | **Lep-10** | **Glu-100 + Lep-5** | **Glu-100 + Lep-10** |
| --- | --- | --- | --- | --- | --- | --- | --- |
| **Control** | - | 0.0108 | 0.0091 | 0.013 | 0.0098 | 0.0083 | 0.0082 |
| **Glu-100** | 0.0108 | - | 0.009 | 0.0412 | 0.0366 | 0.0123 | 0.0098 |
| **Glu-200** | 0.0091 | 0.009 | - | 0.0181 | 0.0418 | 0.006 | 0.0135 |
| **Lep-5** | 0.013 | 0.0412 | 0.0181 | - | 0.009 | 0.0103 | 0.0091 |
| **Lep-10** | 0.0098 | 0.0366 | 0.0418 | 0.009 | - | 0.0029 | 0.011 |
| **Glu-100 + Lep-5** | 0.0083 | 0.0123 | 0.006 | 0.0103 | 0.0029 | - | 0.0494 |
| **Glu-100 + Lep-10** | 0.0082 | 0.0098 | 0.0135 | 0.0091 | 0.011 | 0.0494 | - |

**Table S6C: Statistical comparison of mRNA expression of the Socs1 by *leishmania*-infected THP1 cells treated with leptin and/or glucantime.**

| **Treatment types** | **Control** | **Glu-100** | **Glu-200** | **Lep-5** | **Lep-10** | **Glu-100 + Lep-5** | **Glu-100 + Lep-10** |
| --- | --- | --- | --- | --- | --- | --- | --- |
| **Control** | - | 0.0159 | 0.0028 | 0.0212 | 0.0027 | 0.0003 | 0.0001 |
| **Glu-100** | 0.0159 | - | 0.0335 | ns | 0.0441 | 0.0008 | 0.0002 |
| **Glu-200** | 0.0028 | 0.0335 | - | 0.0339 | ns | 0.017 | 0.0018 |
| **Lep-5** | 0.0212 | ns | 0.0339 | - | 0.045 | 0.001 | 0.0003 |
| **Lep-10** | 0.0027 | 0.0441 | ns | 0.045 | - | 0.0064 | 0.0008 |
| **Glu-100 + Lep-5** | 0.0003 | 0.0008 | 0.017 | 0.001 | 0.0064 | - | 0.0162 |
| **Glu-100 + Lep-10** | 0.0001 | 0.0002 | 0.0018 | 0.0003 | 0.0008 | 0.0162 | - |

**Table S6D: Statistical comparison of protein expression of the Socs1 by *leishmania*-infected THP1 cells treated with leptin and/or glucantime.**

| **Treatment types** | **Control** | **Glu-100** | **Glu-200** | **Lep-5** | **Lep-10** | **Glu-100 + Lep-5** | **Glu-100 + Lep-10** |
| --- | --- | --- | --- | --- | --- | --- | --- |
| **Control** | - | <0.0001 | <0.0001 | 0.0002 | <0.0001 | <0.0001 | <0.0001 |
| **Glu-100** | <0.0001 | - | 0.0016 | 0.0197 | 0.0192 | 0.0006 | 0.0002 |
| **Glu-200** | <0.0001 | 0.0016 | - | 0.0003 | 0.0179 | 0.0006 | <0.0001 |
| **Lep-5** | 0.0002 | 0.0197 | 0.0003 | - | 0.0012 | 0.0003 | 0.0001 |
| **Lep-10** | <0.0001 | 0.0192 | 0.0179 | 0.0012 | - | 0.0001 | <0.0001 |
| **Glu-100 + Lep-5** | <0.0001 | 0.0006 | 0.0006 | 0.0003 | 0.0001 | - | 0.0008 |
| **Glu-100 + Lep-10** | <0.0001 | 0.0002 | <0.0001 | 0.0001 | <0.0001 | 0.0008 | - |

**Table S7A: Statistical comparison of the miR-155 expression by *leishmania*-infected THP1 cells treated with leptin and/or glucantime.**

| **Treatment types** | **Control** | **Glu-100** | **Glu-200** | **Lep-5** | **Lep-10** | **Glu-100 + Lep-5** | **Glu-100 + Lep-10** |
| --- | --- | --- | --- | --- | --- | --- | --- |
| **Control** | - | 0.0084 | 0.0081 | 0.003 | 0.0082 | 0.008 | 0.008 |
| **Glu-100** | 0.0084 | - | 0.009 | 0.0233 | 0.0413 | 0.0105 | 0.009 |
| **Glu-200** | 0.0081 | 0.009 | - | 0.0163 | 0.0363 | 0.0158 | 0.0105 |
| **Lep-5** | 0.003 | 0.0233 | 0.0163 | - | 0.0244 | 0.0094 | 0.0086 |
| **Lep-10** | 0.0082 | 0.0413 | 0.0363 | 0.0244 | - | 0.0125 | 0.0096 |
| **Glu-100 + Lep-5** | 0.008 | 0.0105 | 0.0158 | 0.0094 | 0.0125 | - | 0.009 |
| **Glu-100 + Lep-10** | 0.008 | 0.009 | 0.0105 | 0.0086 | 0.0096 | 0.009 | - |

**Table S7B: Statistical comparison of the miR-146a expression by *leishmania*-infected THP1 cells treated with leptin and/or glucantime.**

| **Treatment types** | **Control** | **Glu-100** | **Glu-200** | **Lep-5** | **Lep-10** | **Glu-100 + Lep-5** | **Glu-100 + Lep-10** |
| --- | --- | --- | --- | --- | --- | --- | --- |
| **Control** | **-** | 0.0142 | 0.0103 | 0.0212 | 0.0117 | 0.0092 | 0.0082 |
| **Glu-100** | 0.0142 | **-** | 0.0113 | 0.0412 | 0.0362 | 0.0169 | 0.0092 |
| **Glu-200** | 0.0103 | 0.0113 | **-** | 0.0195 | 0.0362 | 0.0196 | 0.0109 |
| **Lep-5** | 0.0212 | 0.0412 | 0.0195 | **-** | 0.009 | 0.0125 | 0.0087 |
| **Lep-10** | 0.0117 | 0.0362 | 0.0362 | 0.009 | **-** | 0.0062 | 0.0098 |
| **Glu-100 + Lep-5** | 0.0092 | 0.0169 | 0.0196 | 0.0125 | 0.0062 | **-** | 0.0147 |
| **Glu-100 + Lep-10** | 0.0082 | 0.0092 | 0.0109 | 0.0087 | 0.0098 | 0.0147 | **-** |

**Table S8A: Statistical comparison of SOCS1 expression in *Leishmania*-infected THP-1 cells following treatment with leptin and/or glucantime, analyzed by immunofluorescence.**

| **Treatment types** | **Control** | **Glu-100** | **Lep-10** | **Glu-100 + Lep-10** |
| --- | --- | --- | --- | --- |
| **Control** | - | 0.0016 | 0.0462 | 0.0004 |
| **Glu-100** | 0.0016 | - | 0.0026 | 0.0264 |
| **Lep-10** | 0.0462 | 0.0026 | - | 0.0001 |
| **Glu-100 + Lep-10** | 0.0004 | 0.0264 | 0.0001 | - |

**Table S8B: Statistical comparison of SOCS3 expression in *Leishmania*-infected THP-1 cells following treatment with leptin and/or glucantime, analyzed by immunofluorescence.**

| **Treatment types** | **Control** | **Glu-100** | **Lep-10** | **Glu-100 + Lep-10** |
| --- | --- | --- | --- | --- |
| **Control** | **-** | 0.0013 | 0.0025 | <0.0001 |
| **Glu-100** | 0.0013 | **-** | ns | 0.0079 |
| **Lep-10** | 0.0025 | ns | **-** | 0.0012 |
| **Glu-100 + Lep-10** | <0.0001 | 0.0079 | 0.0012 | **-** |
